# Identify GDPD3 as a key regulator of epithelial–mesenchymal transition and prostate adenocarcinoma progression via the LPA/LPAR1/AKT axis: transcriptomic and experimental study

**DOI:** 10.3389/fimmu.2025.1637325

**Published:** 2026-01-05

**Authors:** Lin Hao, Xiangqiu Chen, Tao He, Tao Wu, Zhiqiang Wen, Ziliang Ji, Xichun Zheng, Qingyou Zheng, Qingchun Zhou, Chengwu He, Qishan Long, Donglin Sun

**Affiliations:** 1 Department of Urology, Shenzhen Hospital, Southern Medical University, Shenzhen, China; 2 Department of Urology, Shenzhen People’s Hospital, The Second Clinical Medical College, Jinan University, Shenzhen, Guangdong, China; 3 The First Affiliated Hospital, Southern University of Science and Technology, Shenzhen, Guangdong, China; 4 Department of Urology, Shenzhen Shockwave Lithotripsy Research Institute, The Eighth Affiliated Hospital of Sun Yat-sen University, Shenzhen, Guangdong, China

**Keywords:** epithelial–mesenchymal transition, GDPD3, prognosis, prostate adenocarcinoma, transcriptomic analysis

## Abstract

**Background:**

Prostate adenocarcinoma (PRAD) is a common malignancy with marked clinical heterogeneity, complicating prognosis and disease monitoring. Traditional tools like the Gleason score lack molecular and microenvironmental insights, underscoring the need for biomarker-driven predictive models.

**Methods:**

Single-cell RNA-seq data from GEO and bulk RNA-seq data from TCGA were analyzed. scRNA-seq processing used the Seurat package, with cluster-specific genes identified via FindAllMarkers. Differentially expressed genes (DEGs) from bulk data were obtained using limma, and key gene modules were identified through WGCNA. Using univariate Cox regression and LASSO analysis, a prognostic model was developed based on cluster-specific genes, key module genes, and differentially expressed genes. Clinical validation included comparison of tumor and adjacent normal tissues, revealing significantly elevated GDPD3 expression, further confirmed by immunohistochemistry. *In vitro* knockdown experiments were conducted in DU145 cells to assess GDPD3’s role in promoting proliferation, migration, and epithelial–mesenchymal transition (EMT).

**Results:**

In this study, through integrated single-cell sequencing and Bulk-RNA-seq analyses, we established a 21-gene prognostic model. QPCR confirmed significant upregulation of three candidates, including GDPD3, which was also elevatedin malignant tissues. Knockdown of GDPD3 inhibited tumor cell proliferation, invasion, and migration. Mechanistically, GDPD3 regulated the levels of lysophosphatidic acid (LPA), which in turn induced EMT in tumor cells. Inhibition or knockdown of the LPA receptor LPAR1 suppressed EMT. LPA promoted EMT through activation of the AKT signaling pathway, and inhibition of this pathway reversed LPA-induced EMT.

**Conclusion:**

This study underscores key molecular mechanisms underlying prostate cancer progression, with GDPD3 emerging as a potential therapeutic target.

## Introduction

Prostate adenocarcinoma (PRAD) is the most common malignancy in males, with its incidence strongly correlated with advancing age (1). In 2020, an estimated 1.4 million new cases of prostate cancer were diagnosed worldwide, contributing to a growing public health burden, particularly in regions with aging populations (1). Radical prostatectomy or radiation therapy are the main curative treatment options for prostate cancer. However, approximately 20% to 30% of patients experience biochemical recurrence after radical prostatectomy, indicating early recurrence and metastasis (2, 3). Currently, several factors have been shown to be effective in predicting patient survival, including serum prostate-specific antigen levels (4), tumor pathological stage, and Gleason score (5). The survival rate of individuals with PRAD is higher when tumors are detected early, and tumor progression significantly impacts patients’ quality of life. Therefore, accurately predicting potential tumor progression is crucial. Over the past decade, the management of prostate cancer has advanced with the incorporation of molecular markers and genetic profiling. Prostate-specific antigen (PSA), Gleason score, and pathologic staging remain fundamental in determining prognosis and guiding treatment decisions. For patients with low-risk disease, active surveillance has become a viable alternative to aggressive treatments, reducing unnecessary side effects while monitoring tumor progression closely. For high-risk patients, androgen deprivation therapy (ADT), combined with chemotherapy or targeted therapies, has been used to control tumor growth and metastasis. Recently, immunotherapies and combination therapies have shown promise, providing new avenues for treating metastatic prostate cancer (2, 3).

Because of the rapid advancement of cancer genetics, Bulk transcriptome sequencing (bulk RNA-seq) has been a prominent tool in transcriptomics in recent decades (4). Genetic changes are increasingly being found as effective therapy targets for PRAD. Wu et al., for example, discovered that targeting the HIC1/TGF-axis-shaped prostate cancer microenvironment slows its growth (5). In addition, according to Hao et al., the deletion of HIC1 has been found to facilitate the spread of prostate cancer through the initiation of epithelial-mesenchymal transition (6). Bulk RNA-seq enhances the comprehension of disease. However, bulk RNA-seq studies only examine the average gene expression in cell groups.

Single-cell RNA-seq (scRNA-seq) now provides access to underlying gene expression distributions and clarifies information on cellular transcriptome heterogeneity (7). With scRNA-seq, it becomes possible to develop potentially beneficial personalized therapeutic approaches for diagnosing cancer throughout its progression. In the study conducted by Yu et al., single-cell omics traces the heterogeneity among prostate cancer cells and the tumor microenvironment, improving our understanding of tumor progression mechanisms (8). Chen et al. also utilized scRNA-seq to determine the correlation between IDH1 expression and the degree of immune cell infiltration, particularly involving immunosuppressive cells like CD8+ T-cells, CD4+ T-cells, and macrophages (9). In light of this advantage, multiple studies have prioritized the identification of possible biological markers for PRAD by integrating bulk RNA-seq and scRNA-seq analyses to effectively stratify and discern patients.

Despite these advances, prostate cancer remains challenging to treat in its advanced stages, and early detection is crucial for improving patient outcomes. Therefore, the identification of novel predictive biomarkers is of paramount importance to enhance personalized treatment strategies. This study utilizes integrative approaches, including bulk RNA-seq and single-cell RNA-seq, to uncover new molecular insights into disease progression and identify potential therapeutic targets for improved clinical management.

## Materials and methods

### The collection and analysis of data

Data from bulk RNA-seq, clinical records, and SNP mutation profiles of TCGA-PRAD were obtained from the TCGA database (https://portal.gdc.cancer.gov/). Clinicopathological information for TCGA-PRAD was acquired from the GDC Data Portal (https://portal.gdc.cancer.gov/projects/TCGA-PRAD) and the TCIA clinical repository (https://www.cancerimagingarchive.net/collection/tcga-prad/). The dataset included 52 normal prostate tissue samples and 492 PRAD tumor samples, all of which contain detailed survival and clinicopathological annotations. The scRNA-seq datasets PRAD_GSE137829, PRAD_GSE141445, and PRAD_GSE172301 were also incorporated into this study. In total, scRNA-seq data from 32 patients with PRAD were analyzed, with all datasets retrieved from the TISCH database (http://tisch.comp-genomics.org/gallery/).

Detailed patient information and sequencing statistics are available in **Table 1**. The integration of samples was performed using the anchors method, a technique implemented in the R “Seurat” (10). Core cells were subsequently created through a filtering step applied to the scRNA-seq data. Unqualified cells refer to genes that exhibit detectability exclusively in three or fewer cells, as well as in less than 200 cells of low quality. Genes identified under these criteria were omitted from subsequent analysis. The analysis of gene expression in core cells involved utilizing a standardized linear regression model, followed by the application of ANOVA to identify the top 2000 genes exhibiting highly variable fingerprints. Principal component analysis (PCA) was conducted on individual cell samples, identifying the first 20 principal components (PCs). These components were then chosen for further study. The UMAP algorithm was employed to comprehensively analyze dimensionality reduction on the initial 20 principal component pairs of the samples (11). R “singleR” was employed to utilize reference datasets such as HumanPrimaryCellAtlasData, BlueprintEncodeData, and ImmuneCellExpressionData for auxiliary annotation (12). Additionally, the CellMarker database and prior research were consulted to identify marker genes for the manual annotation of distinct clusters (13). The visualization process was facilitated by utilizing the R “SCP” (https://github.com/zhanghao-njmu/SCP).

**Table 1:** Basic information of datasets used in this study.

Datasets	Country	No.of patients	No.of controls	Prognostic information
GSE137829	China	6		
GSE141445	China	13		
GSE172301	China	13	–	–
TCGA-PRAD	USA	492	52	Yes

### Gene enrichment analysis based on single-cell marker genes

The Seurat package’s FindAllMarkers function was utilized to locate marker genes for each cluster, with parameters set as min.pct=0.2 and only (14). The Wilcoxon rank sum test was performed to identify differentially expressed genes (DEGs) and screen marker genes when pos=TRUE. Using “clusterProfler” in R software (15), core cells of marker genes underwent enrichment analysis for Gene Ontology (GO) and Kyoto Encyclopedia of Genes and Genomes (KEGG) functions, respectively. ‘Monocle 2’ was utilized to infer the developmental trajectory of cells to elucidate the underlying molecular causes of PRAD progression (16). The “cellchat” algorithm was employed to explore potential cell-to-cell interactions, while “nichenet” was utilized to derive the regulatory links between ligands and immune cell and stromal cell target genes (17, 18).

### DEG identification and functional enrichment analysis in the TCGA-PRAD

A total of 52 control and 492 PRAD patient data were subjected to a difference analysis utilizing the limma package. The criteria of *P* < 0.05 and Log2FC>1 were utilized to identify genes as DEG. The visualization of the heat maps and volcano plots of DEGs was carried out utilizing the ggplot2 and pheatmap packages, respectively. The DEGs were subsequently analyzed by KEGG and GO using the online instrument Metascope (19).

### WGCNA analysis

The “goodSamplesGenes” function, a component of the R “WGCNA”, was employed to assess the necessity of gene filtering within a sample and to determine an optimal soft threshold (20). The analysis involved the establishment of a co-expression network to examine the link between the genes associated with the module (ME) and PRAD.

### The development of a prognosis-predictive model

The identification of candidate genes involved the identification of pairwise intersecting genes from three sources: marker genes of core cells, DEGs associated with PRAD, and module genes. These candidate genes were subsequently used in the least absolute shrinkage and selection operator (LASSO) regression analysis, which was executed using the R “glmnet”. The aim of the LASSO regression was to minimize the number of genes in the final risk model, thereby screening for characteristic genes associated with prognosis (21). In order to decrease the number of genes in the final risk model, variables with *P*-values 0.05 were subjected to the LASSO regression analysis, which was executed utilizing the “glmnet” R package. The prognosis-predictive model was developed employing the subsequent formula: risk score = gene exp11+gene exp22+…+gene expression (where “gene expression” represents the gene expression value and represents the corresponding LASSO regression coefficient). The R programs “survminer” and “ggrisk” were employed to visualize the survival curves and risk maps of patients. Additionally, the “survROC” program was utilized to plot ROC curves, assessing the performance of risk scores in predicting overall survival (OS) at 1, 3, and 5 years among individuals with PRAD.

### Independent prognostication

Univariate analysis was performed to examine the predictive significance of risk models and clinical parameters (age, T stage, M stage, N stage, riskScore). In contrast, multivariable Cox analysis of OS was utilized to determine independent risk factors and predict new events in PRAD. Utilizing the “cph” function in R, a nomogram model was plotted to visualize this predictive model and predict potential new events for patients at 1, 3, and 5 years. The validity of the bar chart was validated through regression analysis (22).

### GSEA and GSVA enrichment analysis

To investigate functional variations and related pathways between the high-risk and low-risk groups, a GSEA was performed utilizing the clusterProfler package on all genes in the TCGA samples from both groups (23). A collection of 50 human cancer marker pathway genes was obtained from the Molecular Signature Database (MSigDB) (https://www.gsea-msigdb.org/gsea/msigdb) (24). Subsequently, GSVA was performed on all genes from samples in both groups, and the variations in GSVA scores between both risk samples were assessed utilizing the “limma” (25, 26).

### Analysis of immune microenvironment

The ssGSEA, implemented through the R “gsva”, was performed for all samples within the TCGA-PRAD dataset to derive profiles of 28 immune cell infiltrations (25). This was done to facilitate the comparison of immune cell characteristics between both risk groups. Pearson’s correlation coefficient was employed to examine the link between risk score and immune infiltrating cells. The R “maftools” was utilized to analyze the mutational variations between both risk groups (27).

### IC50 estimation

The half-maximal inhibitory concentration (IC50) of chemotherapeutic agents was estimated using the *pRRophetic* R package, which is widely applied for drug sensitivity prediction in cancer research. The algorithm constructs a ridge regression model trained on drug response profiles from the Genomics of Drug Sensitivity in Cancer (GDSC) database and predicts the IC50 values of each drug for individual TCGA-PRAD samples based on their bulk RNA-seq expression profiles. All parameters were set to default, and batch correction was performed as recommended in the original pipeline to ensure prediction robustness.

### Human samples

Human tissue samples were collected at Shenzhen Hospital, Southern Medical University under approval from the Institutional Ethics Committee (Approval No.: NYSZYYEC20210011). Written informed consent was obtained from all participants. All samples used in this study were fresh-frozen tissues, which were snap-frozen immediately after collection and stored at –80 °C until further processing.

### Real-time PCR

Total RNA was extracted using RNAiso Plus (TaKaRa), and reverse transcription was performed with an RNA reverse transcription kit (TaKaRa). Actin served as the internal control, and gene expression levels were quantified using the ΔΔCt method. The results were expressed as fold changes compared to the PBS-treated control group. The detailed primer sequences have been included in the **Supplementary Materials** and can be found in the corresponding table (**Supplementary Table 1**).

### Cell line authentication and mycoplasma testing

The DU145 human prostate cancer cell line was obtained from the American Type Culture Collection (ATCC) in February 2025. Cell line authentication was performed prior to experimentation using short tandem repeat (STR) profiling, confirming a match with the reference STR profile and excluding cross-contamination. Mycoplasma contamination was tested using the MycoAlert™ Mycoplasma Detection Kit (Lonza), and all results were negative.

### Seeding and transfection

Cells were seeded in 6-well plates at a density of 2.0–3.0 × 10^5^ cells/well and cultured overnight to reach 60–70% confluence. For plasmid transfection, cells were transfected with 1.5–2.0 μg DNA using Lipofectamine 3000 (Thermo Fisher) according to the manufacturer’s protocol. For siRNA experiments, cells were transfected with 10–30 nM siRNA using Lipofectamine RNAiMAX (Thermo Fisher). All transfections were performed in antibiotic-free medium, followed by replacement with complete medium 24 h later. Cells were collected 24–48 h after transfection for RNA and protein analyses.

### CCK8 assay

Targeted silencing of GDPD3 in DU145 cells was achieved using siGDPD3, while a scrambled siRNA (siNC) was used as a negative control. Cell viability and proliferation were assessed using the Cell Counting Kit-8 (CCK8, Beyotime Biotechnology). Cells were seeded in 96-well plates at a density of 1 × 10^4 cells per well and allowed to adhere for 24 hours at 37°C in a 5% CO2 incubator. After treatment, 10 µL of CCK8 reagent was added to each well, followed by an additional incubation for one to four hours at 37°C (28). The absorbance at 450 nm was measured using a microplate reader (BioTek Instruments) to determine cell viability. To monitor cell growth over time, the assay was conducted at multiple time points (1, 2, 3, and 4 days), and relative cell viability was calculated by normalizing the absorbance values to those of the control group.

### Wound healing assay

The wound healing assay was employed to evaluate cell migration. DU145 cells were transfected with shRNA targeting GDPD3 (shGDPD3) to suppress its expression; shNC served as the control. Cells were seeded onto 6-well plates at a density of 1 × 10^6 cells per well and cultured until they reached 90% confluence. Cells were pretreated with mitomycin-C (10 µg/mL, 2 h) to inhibit proliferation. A linear wound was created in the cell monolayer using a sterile 200 µL pipette tip, and any detached cells were removed by washing the wells twice with PBS. Fresh media, either with or without treatment, was then added, and the cells were incubated at 37°C in a 5% CO2 incubator. Images of the wound area were captured at 0 and 24 hours using a light microscope (×40 magnification). The wound closure rate was calculated by measuring the wound width multiple times using ImageJ software.

### Transwell migration assay

In DU145 cells, GDPD3 expression was knocked down using shGDPD3, with a non-targeting shRNA (shNC) used as the negative control. The Transwell migration assay was conducted using a Transwell chamber (Corning, USA) with an 8 µm pore size to measure cell migration ability (29). Cells were seeded in the top chamber at a density of 5 × 10^5 cells per well in 200 µL of serum-free media. The lower chamber contained 600 µL of complete medium with 10% FBS as a chemoattractant. After a 24-hour incubation, non-migratory cells on the top surface of the membrane were removed with a cotton swab. The migrated cells on the bottom surface were fixed with 4% paraformaldehyde for 15 minutes and stained with crystal violet for 30 minutes. Images of the stained cells were taken using a light microscope (×40 magnification), and the number of migratory cells in five random fields was counted.

### LPA measurement

LPA levels were quantified using a commercial ELISA kit (Invitrogen, EEL166) following the manufacturer’s protocol. Briefly, cell culture supernatants were collected, clarified by centrifugation, and incubated in LPA-specific antibody-coated wells. Absorbance at 450 nm was measured using a microplate reader, and LPA concentrations were calculated from a standard curve.

### Immunofluorescence

Cells were fixed with 4% paraformaldehyde for 15 min, permeabilized using 0.1% Triton X-100 for 10 min, and blocked with 5% BSA for 1 h at room temperature. Cells were then incubated overnight at 4°C with the anti-GDPD3 primary antibody (Abcam, ab214375), followed by incubation with fluorophore-conjugated secondary antibodies for 1 h at room temperature in the dark. Nuclei were counterstained with DAPI, and fluorescence images were acquired using a Leica fluorescence microscope.

### Western blot

Proteins were extracted from cells or tissues using lysis buffer containing PMSF, and the concentration was determined using a BCA kit. Protein samples (20μg per lane) were mixed with 5× loading buffer and boiled for 10 min. Proteins were separated on 10% SDS–polyacrylamide gels, followed by transfer onto PVDF membranes (Millipore). The membrane was blocked with 5% skim milk in TBST for 2 hours, then incubated with primary antibody (1:1000) overnight and secondary antibody (1:5000) for 1 hour, with three washes in TBST between incubations. Finally, the proteins were detected using chemiluminescent substrate (ECL) and imaged with a chemiluminescence imaging system. Primary antibodies, including E-cadherin (Proteintech, 20874-1-AP), Vimentin (Proteintech, 10366-1-AP), α-SMA (Proteintech, 14395-1-AP), LPAR1 (Proteintech, 86655-2-RR), AKT (Proteintech, 10176-2-AP), phospho-AKT (p-AKT; Proteintech, 66444-1-Ig), and β-Actin (Proteintech, 66009-1-Ig) as a loading control.

### Statistical analysis

R software (version 4.2.1) was used for all further data processing and statistical analyses. The methodologies for the bioinformatics assessments are detailed in the relevant subsections. Statistical significance was denoted as follows: * p < 0.05, ** p < 0.01, and *** p < 0.001. A p-value below 0.05 indicates significance.

## Results

### Single-cell RNA sequencing analysis and functional enrichment in PRAD patients

**Figure 1:**
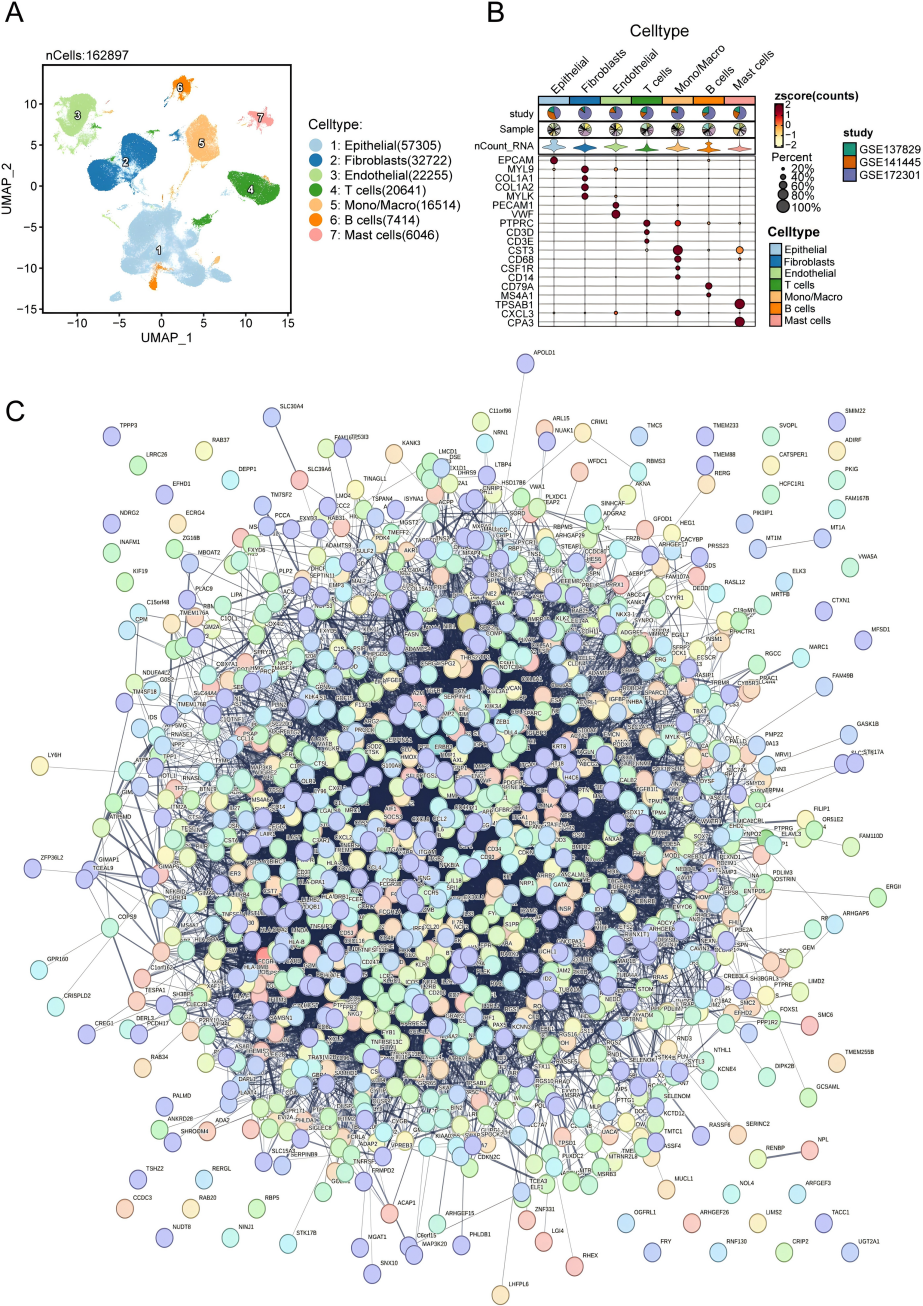
Single-cell transcriptomic profiling identifies major cell types and their signature genes in PRAD. **(A)** UMAP visualization of cell populations in the PRAD samples. **(B)** Dot plot showing the marker gene across different cell type. **(C)** PPI enrichment network.

The fundamental framework of the current research is depicted in **Supplementary Figure S1**. Initially, a collection of scRNA-seq samples was obtained from a database consisting of 32 patients diagnosed with PRAD. Subsequently, a rigorous screening process was conducted to eliminate cells that did not meet the predetermined criteria. As a result, a total of 162,897 cells were successfully retrieved and deemed suitable for subsequent investigations. The annotation of cell clusters was executed using the CellMarker database (30), and relevant references, generating seven cell clusters (**Figure 1A**). The cell types present include fibroblasts, endothelial cells, epithelial cells, monocyte/macrophages, B cells, T cells, and mast cells. Bubble charts were utilized to visually represent the expression of critical marker genes for each cell type (**Figure 1B**). Subsequently, the feature genes associated with cell types were subjected to GO and KEGG enrichment analyses. The majority of these pathways were enriched in tumor- and immune-related pathways (**Supplementary Figures S2A–G**). Then, we performed PPI network analysis on the feature genes of these cell types to identify key genes associated with PARD (**Figure 1C**).

Furthermore, Pseudo-time trajectory analysis using Monocle 2 revealed three distinct differentiation pathways in PRAD, demonstrating a gradual progression from progenitor states. Notably, immune and non-immune cells exhibited temporally divergent differentiation patterns: non-immune lineages predominantly underwent early-stage specification, while immune cell maturation was biased toward later trajectory phases (**Supplementary Figure S3A**). The usage of ligand-receptors is expected to facilitate intercellular communication. The data showed that fibroblasts, endothelial cells, and epithelial cells communicated more often (**Supplementary Figure S3B**). Moreover, it was observed that the MIF cell signaling pathway exhibited significant activity in facilitating intercellular communication. The major ligand pairs involved in the MIF signaling pathway are CD74-CXCR4 and CD74-CD44 (**Supplementary Figure S3C**). Non-immune cells are primarily responsible for generating MIF signals, while immune cells consist of signal receptors and influences (**Supplementary Figures S3D, E**). Furthermore, our analysis of the regulatory network for ligands and target genes indicated that 28 ligands were selectively activated in immune cells (**Supplementary Figure S3F**). A heat map was generated to visualize the distribution of 28 ligands on immune cells and their impact on the regulation of target genes in non-immune cells. (**Supplementary Figure S3G**).

In summary, we identified the characteristic genes of different cell types and highlighted their functions.

### Identification and functional enrichment analysis of DEGs in bulk RNA-seq

**Figure 2:**
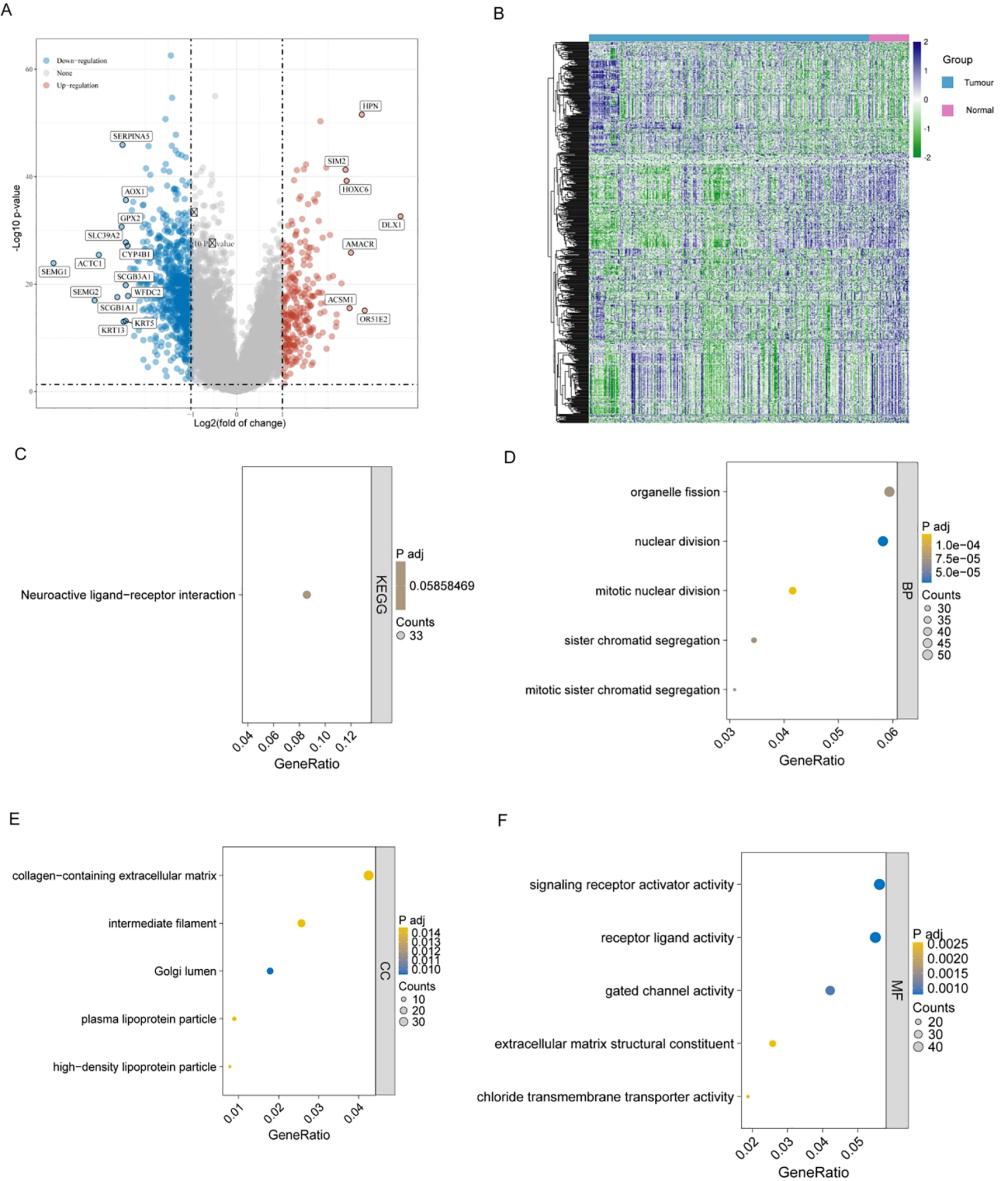
Differential expression analysis reveals transcriptomic alterations between PRAD tumors and normal tissues. **(A)** DEGs volcano diagram comparing PRAD and control in TCGA. Significant DEGs were identified with P<0.05 and |log2FoldChange|>1. Red dots show elevated genes, and blue dots show genes with reduced expression. **(B)** Heatmap of DEGs. **(C–F)** Bar plots of the GO and KEGG analysis of DEGs.

In the TCGA-PRAD database, we analyzed the differentially expressed genes (DEGs) between the tumor and normal groups. The corresponding clinical information used in this analysis has been provided in the **Supplementary Materials** (**Supplementary Table 2**). A total of 1,219 significant DEGs were identified. The distribution of DEGs was visually represented using volcano plots and heatmaps (**Figures 2A, B**). Then, we conducted KEGG and GO enrichment analyses of DEGs. The KEGG analysis revealed that these DEGs were primarily enriched in the neuroactive ligand-receptor interaction pathway (**Figure 2C**). The GO analysis showed that the DEGs were mainly enriched in pathways associated with various tumor-related biological processes (**Figures 2C–F**).

### Identification of key modules related to PRAD

**Figure 3:**
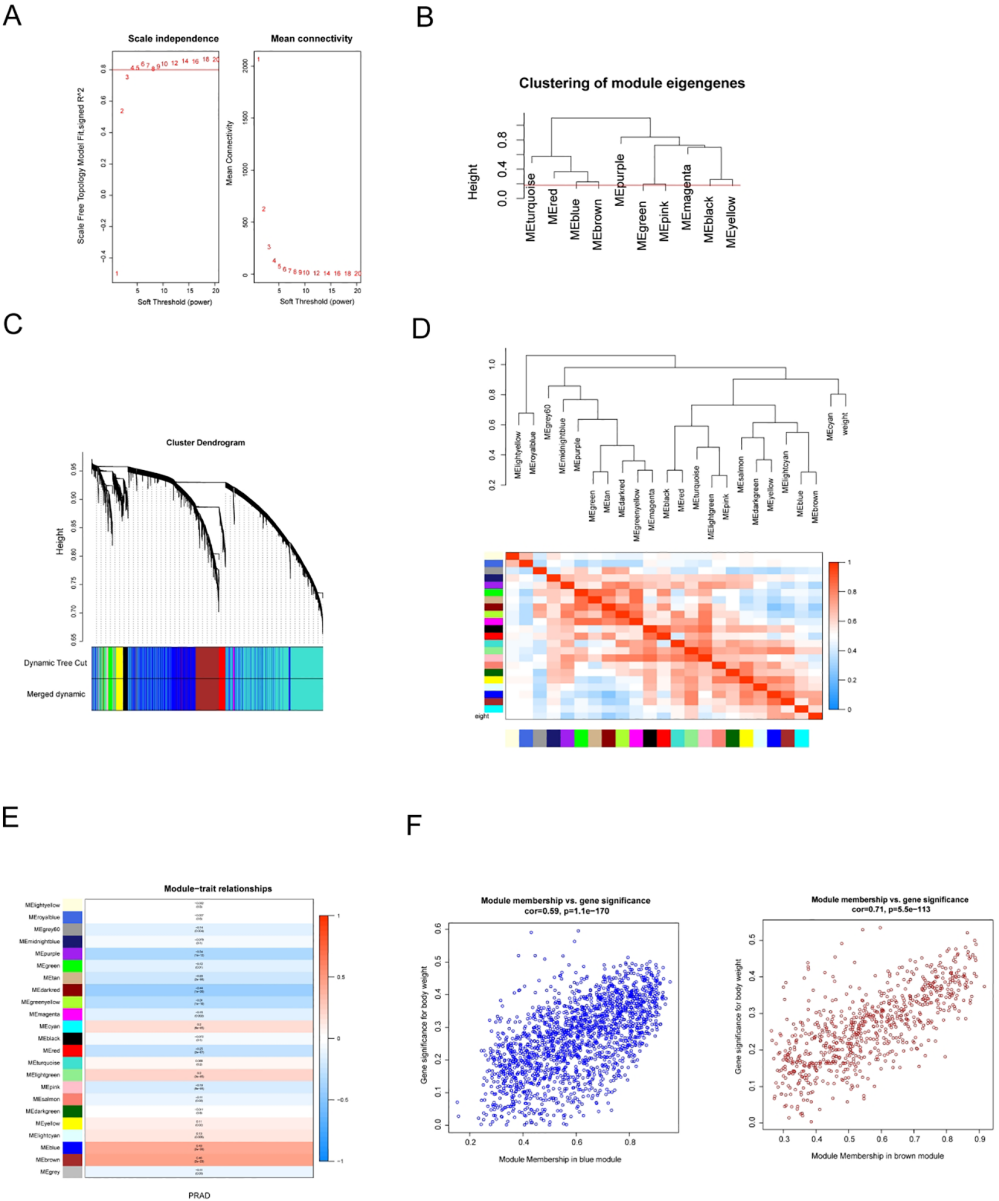
WGCNA identifies PRAD-related gene modules and key regulatory clusters. **(A)** Evaluation of the scale-free index for different soft-threshold powers (β). **(B)** The minimum number of genes per module is 300, and 23 modules are obtained when MEDissThres is equal to 0.18. **(C)** The cluster dendrogram representing co-expression network modules (1-TOM). **(D)** A heatmap depicting the relationships between the identified modules. **(E)** Analysis of correlations between modules along with corresponding P-values. **(F)** Scatter plot analysis focusing on the brown and blue modules.

WGCNA was utilized to identify participants in the progression and development of PRAD. While constructing the co-expression network, the power was set to 5 when the fit index of the soft-threshold scale-free topology attained 0.80 (**Figures 3A, B**). In order to integrate a similar module tree algorithm through dynamic shear analysis, MEDissTres was set to 0.2. Following the merging process, ten modules were obtained (**Figure 3C**), and a correlation heatmap depicting the relationships between these modules was generated (**Figure 3D**). As per the correlation coefficient and *P*-value, MEbrown and MEblue (consisting of 2545 genes) were chosen as the key modules (**Figure 3E**). A scatter plot was generated to illustrate the clinical relevance of the brown and blue modules (**Figure 3F**).

### Construction of a three-characteristic gene-based model

**Figure 4:**
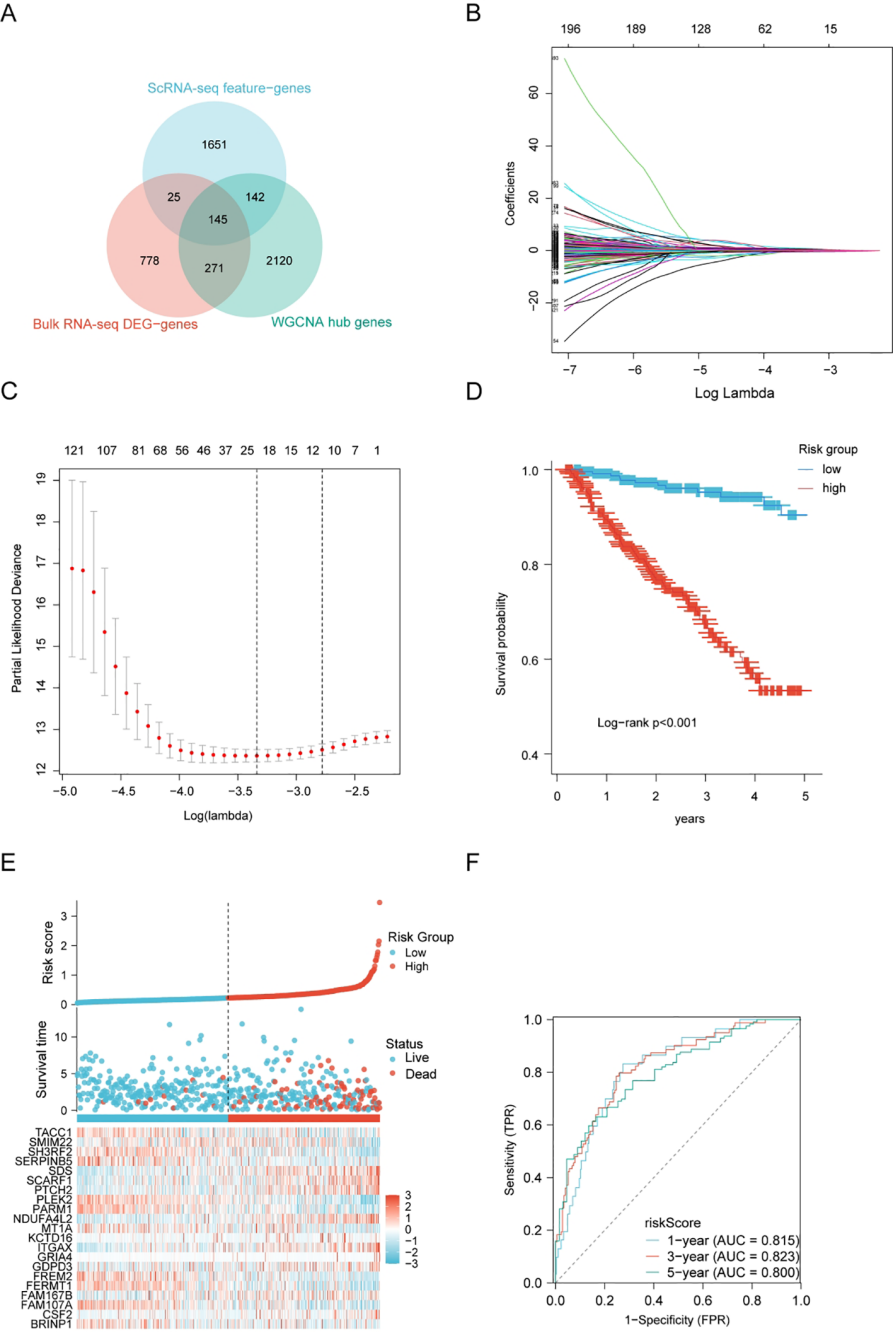
Construction of a prognostic gene signature for PRAD. **(A)** Overlap of feature genes, WGCNA, and DEGs in PRAD and controls. **(B, C)** LASSO regression of OS-related genes. **(D)** Kaplan–Meier curve results. **(E)** Risk survival status plot. **(F)** Assessment of the AUC for predicting 1, 3, and 5-year survival rates among PRAD individuals.

Using Venn diagrams, we illustrated the intersection of feature genes from single-cell sequencing, WGCNA module genes, and DEGs of cell Bulk RNA-seq. A total of 145 overlapping genes were identified (**Figure 4A**). Then, we performed Lasso regression to select the key genes (**Figures 4B, C**). Subsequently, a riskScore model was constructed based on the expression of 21 risk-associated genes (**Table 2**). The Kaplan-Meier indicated a statistically significant difference in OS between individuals with high-risk and low-risk scores (**Figures 4D, E**). To enhance the credibility of the risk model, an assessment was conducted by calculating the Receiver Operating Characteristic (ROC) curve for OS. The Area Under the Curve (AUC) values at 1, 3, and 5 years were found to be larger than 0.80, suggesting a higher level of effectiveness of the risk model (**Figure 4F**). In conclusion, our prognostic model demonstrated outstanding predictive accuracy for PRAD. To evaluate the robustness of our risk model, we validated it using an external dataset from the ICGC database. The Kaplan–Meier survival analysis showed that patients in the high-risk score group had a significantly higher mortality rate, which is consistent with the results of our risk model (**Supplementary Figure S7A**). Furthermore, we analyzed the prognostic relevance of the 21 risk-related genes in PRAD and found that the majority of these genes were negatively correlated with patient prognosis (**Supplementary Figure S7B**). These findings support the predictive accuracy of the risk score model based on these genes in PRAD.

**Table 2:** The formula for riskScore.

Gene	Coefficient (β)
CSF2	0.8071
BRINP1	-0.0166
FAM107A	-0.073
FAM167B	0.0189
FERMT1	-0.0554
FREM2	-0.4522
GDPD3	0.0182
GRIA4	0.4062
ITGAX	0.0430
KCTD16	0.1516
MT1A	-0.0872
NDUFA4L2	0.0910
PARM1	-0.0935
PLEK2	-0.4064
PTCH2	0.5900
SCARF1	0.3935
SDS	0.1416
SERPINB5	-0.0027
SH3RF2	-0.0044
SMIM22	-0.0671
TACC1	-0.0565

### Evaluation of independent prognosis-predictive factors and nomogram development

**Figure 5:**
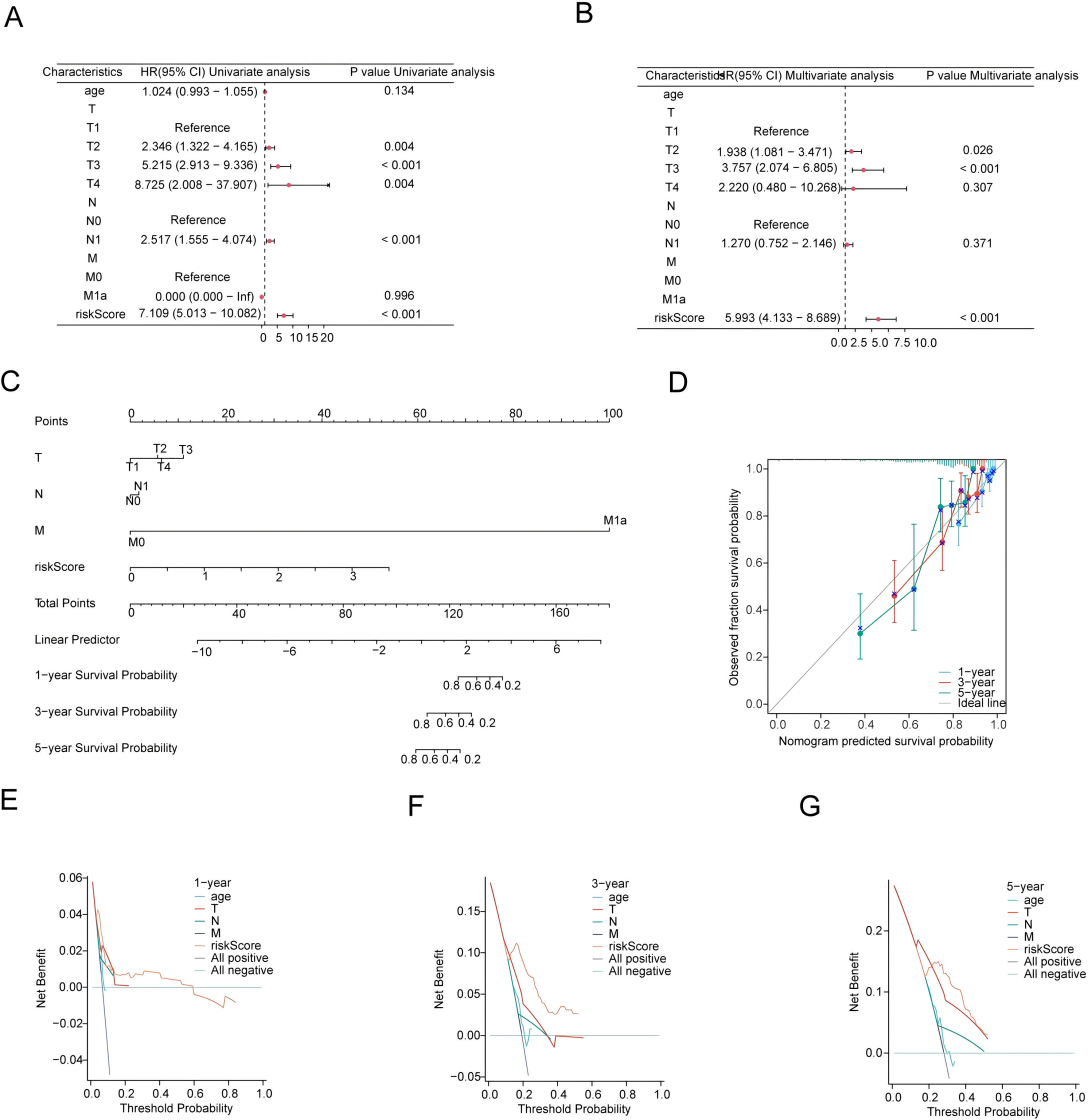
Establishment and validation of a prognostic nomogram model. **(A)** A Univariate Cox analysis involving risk scores and clinical characteristics. **(B)** Multifactorial Cox analysis. **(C)** Development of the nomogram model. **(D)** Representation of the calibration curve of the nomogram. **(E-G)** Decision curve analyses assessing the performance of nomogram for 1-, 3- and 5-year risk predictions.

The study employed univariate and multivariate Cox analyses to determine independent predictive factors to assess clinical features and risk scores. RiskScore demonstrated superior predictive capability compared to the T, M, and N stages. (**Figures 5A, B**). The nomogram model (**Figure 5C**) incorporated the independent prognostic variables. Furthermore, the calibration curve demonstrated the substantial predictive efficacy of the model (**Figure 5D**). The accuracy of 1-, 3-, and 5-year predictions for was assessed using the Net benefit strategy (**Figures 5E–G**). Consequently, the findings of the study revealed that risk score served as an independent prognosis-predictive factor and that the nomogram exhibited a high predictive capability for DFS in individuals with PRAD.

### GSEA and mutations analysis between high− and low-risk groups

To explore the influence of both risk groups on cancer advancement, GSEA was conducted to identify the most significant enrichment pathways distinguishing between the two. The findings indicated that the high-risk group exhibited significant enrichment in immunological processes, including TNF-α and Interferon-α pathways, whereas the low-risk group exhibited enrichment primarily in androgen and acid metabolism pathways (**Supplementary Figures S4A–C**). Furthermore, GSVA was performed on all genes in both risk groups. The findings indicated that the high-risk group exhibited significant expression in processes related to cell communication, cyclase regulation, and cyclic nucleotide phosphodiesterase. Moreover, the low-risk group exhibited activation in the areas of tyrosine kinase, biosynthesis, and metabolism (**Supplementary Figure S4D**). It was discovered that missense mutations and SNPs dominated the PRAD patients (**Supplementary Figure S4E**). The mutation analysis outcomes between both risk groups revealed that the majority of mutation types in both groups were missense mutations. The high-risk group exhibited a larger percentage of mutations in comparison to the low-risk group (**Supplementary Figures S4F, G**). Overall, the findings of this study highlighted that predicted genes, pathways, and site mutations are relevant in PRAD.

### Immune infiltration analysis

The ssGSEA methodology was utilized to calculate infiltration scores for 28 distinct immune cell populations across various risk groups. The findings unveiled statistically significant differences in infiltration levels among 17 immune cell species (**Supplementary Figure S5A**). The heatmap illustrates the correlation between prognostic genes and immune cells (**Supplementary Figure S5B**). The CIBERSORT algorithm was used to assess the correlation between riskScore and tumor-infiltrating immune cells (**Supplementary Figure S5C**), while the ESTIMATE algorithm was employed to evaluate the immune and stromal components of the tumor microenvironment (**Supplementary Figure S5D**).

### Sensitivity analysis of potential clinical drugs within groups identified as high-risk and low-risk

We assessed the drug sensitivity of 6 potential drugs in PRAD patients by comparing their IC50 values, and our findings revealed that the high-risk cohort demonstrated a greater responsiveness to MTX, 5-FU, ADM and DDP in comparison to the low-risk cohort. Interestingly, patients with low-riskScore displayed an elevated sensitivity to CTX (**Supplementary Figures S6A–F**). Our study indicated that riskScore critically involved in the regulation of immune checkpoints, including CD274, PDCD1LG2, and IGSF8, were found to be expressed at elevated levels within the high-risk cohort, while CTLA4, HAVCR2, LAG3, PDCD1, TIGIT, and ITPRIPL1 showed higher expression in the low-risk group. These findings may provide valuable insights for immunotherapy (**Supplementary Figure S6G**).

### Identifies GDPD3 as a key mediator of PRAD cell invasiveness

**Figure 6:**
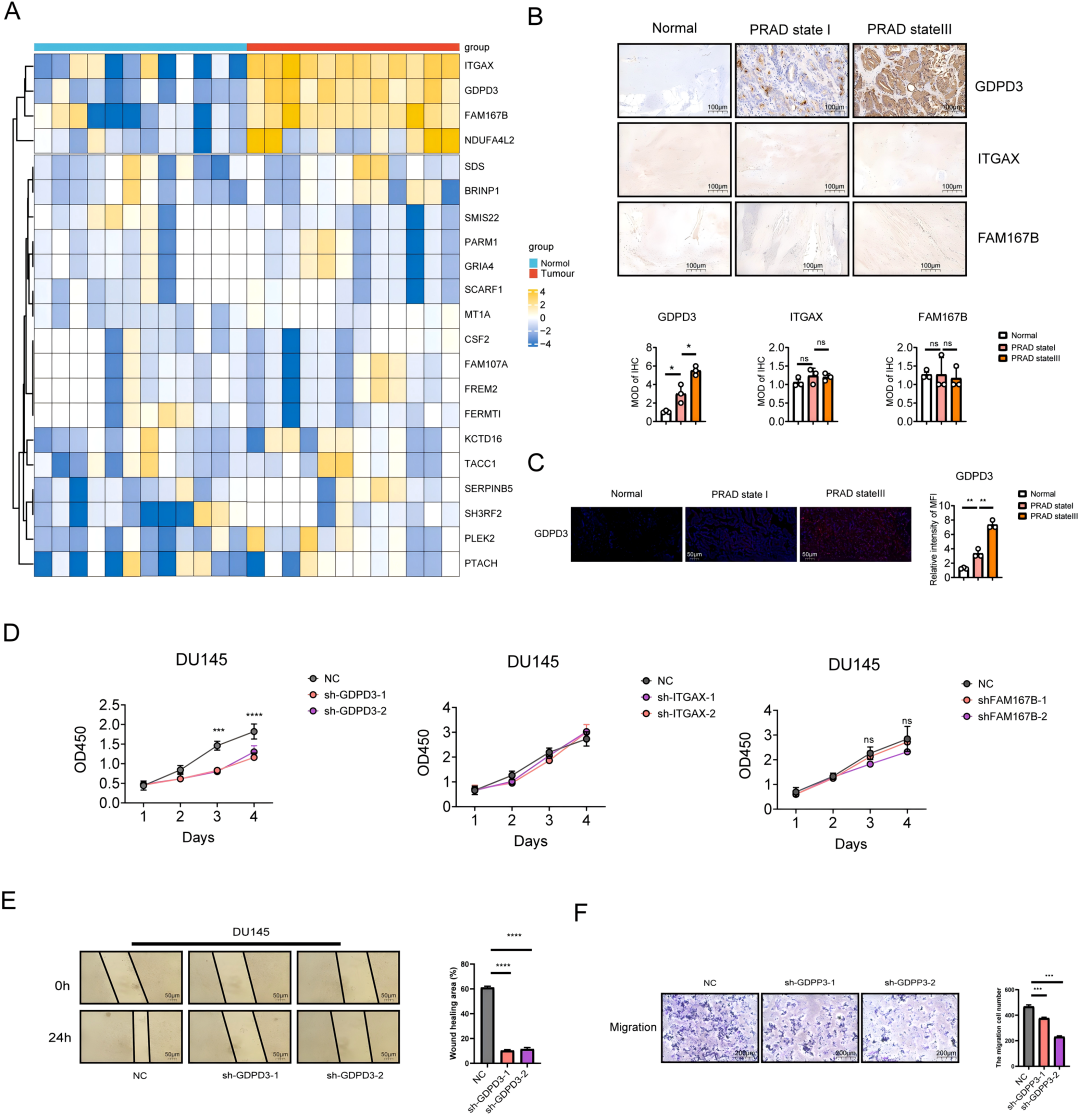
Expression and functional validation of risk genes in PRAD. **(A)** Detect the mRNA expression of the 21 model genes in PRAD tumors and normal tissues. **(B)** Immunohistochemical detection of the risk genes GDPD3, ITGAX, and FAM167B. Scale bar 100 μm. **(C)** Examine the expression of GDPD3 by immunofluorescence across different tumor stages. Scale bar 50 μm. **(D–F)** Cell proliferation, migration, and invasion were assessed following knockdown of GDPD3, ITGAX, or FAM167B using shRNAs. Cell proliferation scale bar is 50 μm. Proliferation was measured using the CCK-8 assay, while migration and invasion were evaluated using Transwell assays. *p < 0.05, **p < 0.01, ***p < 0.001, ****p < 0.0001.

In order to validate the accuracy of our analysis, we collected 12 tumor tissues samples from PRAD patients. 12 normal samples were used as controls. First, we detected the mRNA expression of 21 risk genes and found that GDPD3, ITGAX, and FAM167B were significantly overexpressed in tumor tissues (**Figure 6A**). We further analyzed the expression patterns of these three genes at the single-cell level. GDPD3 and FAM167B were predominantly expressed in progenitor cells, whereas ITGAX was mainly expressed in monocyte–macrophage populations (**Supplementary Figures S8A–I**). Subsequently, we examined the protein expression of all three genes and found that GDPD3 was highly expressed in tumor tissues, with levels increasing progressively across clinical stages. In contrast, ITGAX and FAM167B showed no significant differences in expression. (**Figure 6B**). These findings were further confirmed through immunofluorescence analysis (**Figure 6C**). Subsequently, *in vitro* experiments were conducted using DU145 cells transfected with shRNAs targeting GDPD3, ITGAX, and FAM167B to assess their effects on cell proliferation, migration, and invasion. Knockdown of GDPD3 significantly reduced all three cellular functions, whereas silencing of ITGAX and FAM167B showed no significant effects (**Figures 6D, E**). To further validate the functional relevance of the candidate genes, we employed the LNCaP prostate cancer cell line as an additional model system. Among the genes examined, only GDPD3 knockdown produced a substantial phenotypic effect in LNCaP cells. Silencing GDPD3 significantly reduced cell proliferation and markedly impaired migration and invasion, as evidenced by the pronounced decrease in transwell‐traversing cells. In contrast, knockdown of the other genes did not result in observable changes in any of these phenotypes. These results confirm that GDPD3 is the only gene among those tested that is required to maintain the proliferative, migratory, and invasive capacities of LNCaP prostate cancer cells (**Supplementary Figures S9A–E**).These results identify GDPD3 as a key regulator of prostate cancer cell proliferation, migration, and invasion, whereas ITGAX and FAM167B appear to have limited functional impact.

### GDPD3 promotes EMT via LPA–LPAR1 signaling

**Figure 7:**
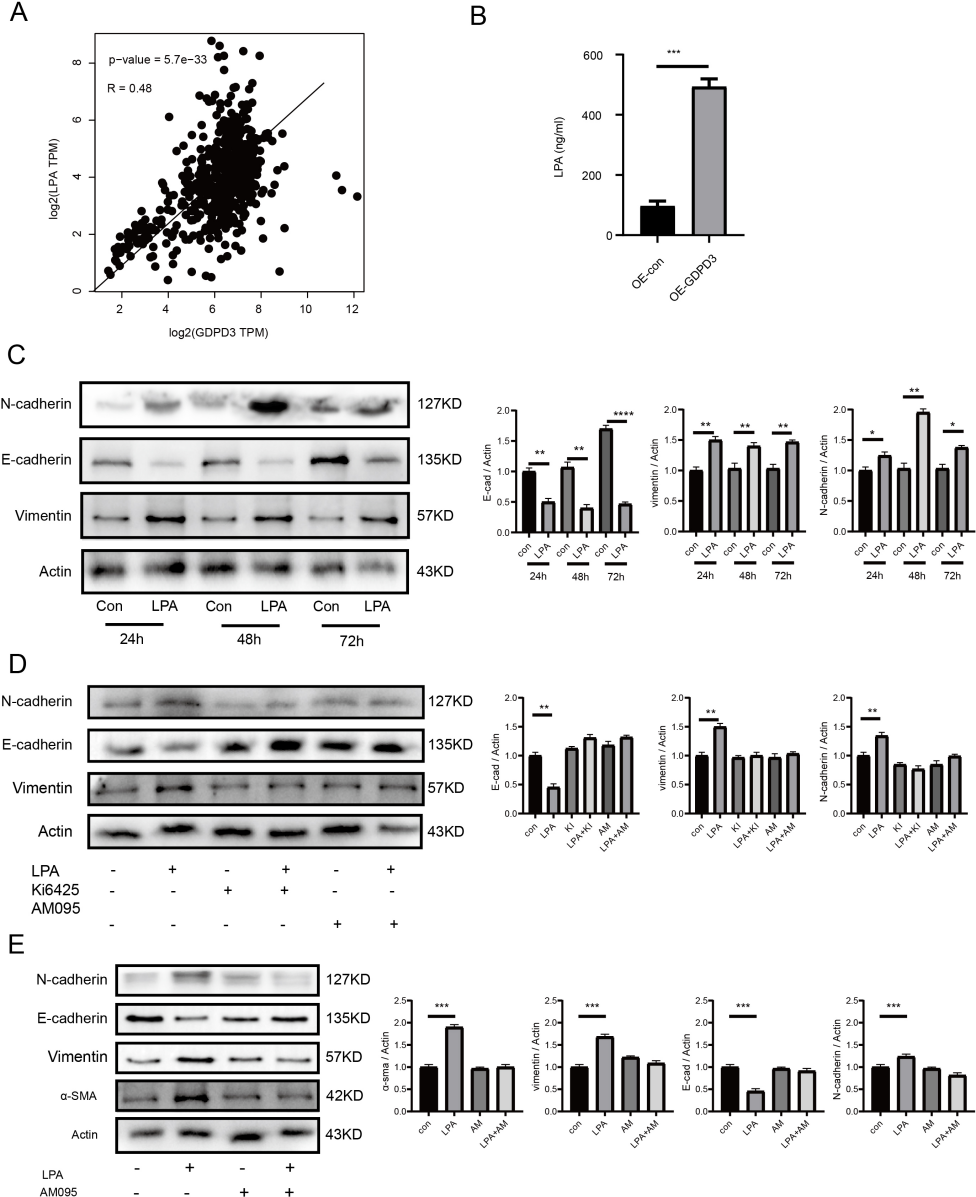
GDPD3 promotes EMT through LPA–LPAR1 signaling. **(A)** Analysis of the correlation between GDPD3 and LPA. **(B)** LPA levels were measured by ELISA following GDPD3 overexpression in DU145 cells. **(C)** DU145 cells were treated with either vehicle control or 20 μM LPA for 24, 48, and 72 hours. Protein levels of N-cadherin, E-cadherin and Vimentin were assessed by Western blot and quantified. **(D, E)** DU145 cells were treated with LPA alone, or with LPA in combination with KI6425 or AM095 (both LPAR1 inhibitors). EMT markers N-cadherin, Vimentin and α-SMA were evaluated by Western blot, and band intensities were quantified using ImageJ software, normalized to actin. (n = 3–4 independent experiments). Data are presented as mean ± SEM. *p < 0.05, **p < 0.01, ***p < 0.001, ****p < 0.0001.

Given that GDPD3 encodes a glycerophosphodiesterase involved in lysophospholipid metabolism (31), we investigated whether its upregulation influences LPA production. First, we analyzed the correlation between GDPD3 and LPA in the TCGA-PRAD dataset and found a strong positive association (**Figure 7A**). To further investigate, we overexpressed GDPD3 and observed an increase in LPA levels (**Figure 7B**). We then explored whether LPA could induce epithelial–mesenchymal transition. DU145 cells were treated with LPA for 24, 48, and 72 hours, and EMT induction was detected as early as 24 hours (**Figure 7C**). Next, we used the LPAR1 inhibitors Ki6425 and AM095 to block LPA signaling. Inhibition of LPAR1 led to decreased expression of EMT markers Vimentin and α-SMA (**Figures 7D, E**). LPA stimulation selectively increased the mRNA expression of Twist, thereby enhancing the EMT program, while the levels of Slug and Snail remained unchanged (**Supplementary Figures S10A, B**). Conversely, genetic deletion of LPAR1 significantly reduced Twist expression without affecting Slug or Snail, indicating that LPA promotes EMT primarily through an LPAR1–Twist axis(**Supplementary Figures S10C, D**). These findings suggest that GDPD3 promotes epithelial–mesenchymal transition in prostate cancer cells by elevating LPA levels, which activate downstream signaling through LPAR1.

### LPAR1 mediates LPA-induced EMT via AKT signaling

**Figure 8:**
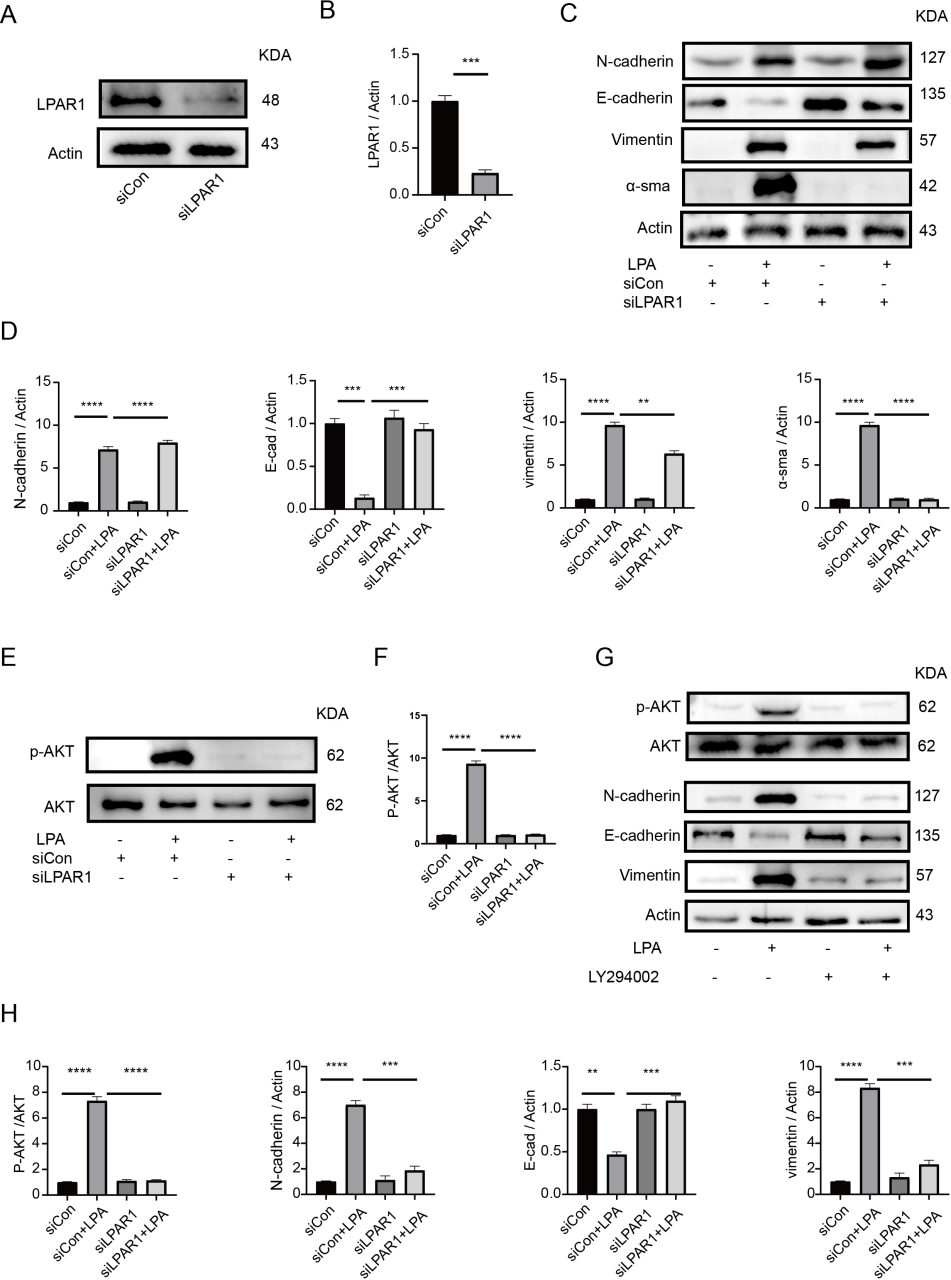
LPAR1 knockdown and AKT inhibition attenuate LPA-induced EMT. **(A, B)** LPAR1 was knocked down, and knockdown efficiency was subsequently validated. **(C, D)** DU145 cells were transfected with siRNA targeting LPAR1, and the expression levels of N-cadherin, E-cadherin and Vimentin were assessed by Western blot. **(E, F)** Western blot analysis was performed to assess the expression levels of AKT and phosphorylated AKT (p-AKT). **(G, H)** DU145 cells were treated with 2 μM LY294002 (AKT pathway inhibitor), and the protein levels of AKT, phosphorylated AKT (p-AKT), N-cadherin, E-cadherin and Vimentin were assessed by Western blot and quantified. Band intensities were quantified using ImageJ software and normalized to actin. *p < 0.05, **p < 0.01, ***p < 0.001, ****p < 0.0001.

To further validate the role of LPAR1 in LPA-mediated epithelial–mesenchymal transition (EMT), we performed siRNA-mediated knockdown of LPAR1 (**Figures 8A, B**). Silencing of LPAR1 markedly suppressed EMT, as evidenced by increased E-cadherin and decreased Vimentin expression (**Figures 8C, D**). Mechanistically, LPA treatment induced phosphorylation of AKT, whereas LPAR1 knockdown reduced this phosphorylation, indicating that LPA activates AKT signaling via LPAR1(**Figures 8E, F**). Moreover, treatment with an AKT pathway inhibitor attenuated LPA-induced EMT, further supporting the involvement of the LPA–LPAR1–AKT axis in regulating EMT(**Figures 8G, H**). These findings reinforce that GDPD3-driven LPA production promotes EMT in prostate cancer cells through LPAR1-dependent activation of AKT signaling.

## Discussion

PRAD is one of the most frequent cancers globally, and its prevalence is increasing in many nations (32). Despite recent efforts to improve PRAD care, the varied and aggressive aspects of PRAD remain limited for prognostic evaluation (33). Thus, the screening of novel biomarkers to aid in the development of patient-specific therapies and enhance prognosis remains crucial and imperative. Unlike bulk RNA-seq, which provides insights into the average gene expression levels across cell populations, scRNA-seq has emerged as a valuable technique for transcriptional stratification. By delineating cell subpopulations and identifying specific biomarkers, scRNA-seq enables the characterization of heterogeneity among distinct cell types in multiple cancer types, including PRAD. Consequently, this investigation executed a comprehensive analysis of both bulk RNA-seq and scRNA-seq data, culminating in the development of a risk model that exhibits outstanding prognosis-predictive value in the context of PRAD.

Initially, an in-depth investigation was conducted using scRNA-seq data comprising 162,897 cells to identify seven crucial cell types present in the PRAD tumor. These cell types included epithelial cells, fibroblasts, endothelial cells, T cells, monocyte macrophages, B cells, and mast cells. Remarkably, a high cellular communication frequency was observed among these cell types. Notably, our analysis detected the substantial activation of the MIF signaling pathway during the progression of PRAD. Interestingly, similar findings have been reported in colorectal cancer (CRC), where the MIF/CD74 pathway has been implicated in regulating disease progression (34). In PRAD, it was further revealed that the CD74-CXCR4 axis plays a pivotal role in this abnormally active ligand-receptor interaction within the MIF pathway. These findings suggested a potential parallel between CRC and PRAD, whereby CD74 may be critically involved in promoting the onset and advancement of PRAD. The functional analysis of the DEGs identified in the TCGA dataset revealed significant enrichment in the NABA MATRISOME ASSOCIATED signaling pathway and the MITOTIC CELL CYCLE PROCESS signaling pathway (35). These pathways are believed to contribute to the proliferation and advancement of PRAD. Prior research has consistently reported alterations in cyclins, TP53, and Rb genes, which are known to be involved in PRAD (36).

Additionally, the expression of Amphetamine has been observed in various diseases, particularly in epithelial cells (37). This observation may be associated with the more infiltrative nature of PRAD epithelial cells. Moreover, through the application of WGCNA analysis, the brown and blue modules, comprising 2,120 genes, were identified as key modules. By intersecting the three aforementioned gene sets, a total of 145 candidate genes were collected to enhance the stability of the gene signature.

Subsequently, a gene prognosis-predictive model was constructed utilizing univariate Cox regression analysis and the LASSO algorithm (38). The ROC curve demonstrated that the model exhibited promising predictive efficacy for prognosis in PRAD. Furthermore, the results of this study indicated that this gene prognostic model functioned as an independent predictive factor for OS among PRAD patients. Distinguished from other models, our signatures were derived through the integration of diverse datasets and utilizing various algorithms. These signatures underwent rigorous validation using an independent training set to ensure robustness. Remarkably, our model exhibited impressive AUC values ranging from 0.800 to 0.823, further highlighting its superior performance and reliability.

Additionally, all samples were categorized into low- and high-risk groups based on the calculated risk score. The observations in this research unveiled that the high-risk group exhibited significant enrichment in immune processes and immune-related pathways. This led to the hypothesis that the risk score could function as a potential prognostic marker for individuals with PRAD undergoing immunotherapy. To further explore this hypothesis, multiple aspects were examined, including tumor mutational burden (TMB), immune infiltration, and associated risk genes. The comprehensive analysis revealed that the high-risk group exhibited higher TMB and remarkably increased levels of immune cell infiltration. These outcomes collectively indicate that the risk score holds promise as a valuable tool for predicting the response to immunotherapy in individuals with PRAD. Our immune-related analysis revealed that the riskScore is positively correlated with key immune checkpoint molecules such as CD274, PDCDILG2, and IGSF8. This correlation suggests that riskScore may be involved in modulating the tumor immune microenvironment, potentially facilitating immune escape mechanisms in cancer cells.

Among the 21 prognostic genes identified through integrative transcriptomic analysis, GDPD3 emerged as a particularly compelling candidate due to its consistent upregulation in tumor tissues and its significant association with poor clinical outcomes. Building on this observation, we conducted a series of functional and mechanistic studies to elucidate the role of GDPD3 in prostate cancer progression. Our results demonstrate that GDPD3 facilitates epithelial–mesenchymal transition (EMT) by increasing intracellular levels of lysophosphatidic acid (LPA), which subsequently activates the LPAR1–AKT signaling pathway. This axis promotes a mesenchymal phenotype, as evidenced by downregulation of E-cadherin and upregulation of Vimentin. These findings not only confirm GDPD3 as a functional driver of malignancy but also establish a mechanistic link between lipid metabolism and tumor cell plasticity, paving the way for targeted therapeutic strategies.

## Limitations

This study has several limitations. Firstly, the relatively small sample size used for validation may not fully capture the tumor heterogeneity in the broader prostate cancer population, requiring further verification in larger cohorts. Additionally, the use of a single prostate cancer cell line, DU145, may introduce cell line–specific biases, and future studies should incorporate multiple cell lines to ensure the generalizability of the findings. Moreover, while both bulk RNA-seq and single-cell RNA-seq provided valuable insights, they carry inherent constraints, such as signal averaging in bulk RNA-seq and sequencing-depth limitations in single-cell RNA-seq (39). We also recognize that Gleason- or pathology-level stratification was not incorporated in the current study. This decision was primarily driven by the limited sample size and the lack of uniformly annotated pathological data. Nonetheless, we acknowledge the importance of such analyses and have added a clear statement in the Discussion section to clarify this limitation and the rationale behind it. Lastly, while we explored the role of GDPD3 in LPA/LPAR1/AKT signaling, further mechanistic studies are needed to fully elucidate its contribution to prostate cancer progression.

## Conclusions

In this study, we integrated single-cell and bulk transcriptomic data to construct a 21-gene prognostic model for prostate adenocarcinoma (PRAD), identifying GDPD3 as a key gene associated with tumor progression. Clinical validation confirmed its upregulation in tumor tissues, and functional assays demonstrated that GDPD3 promotes cell proliferation, migration, and invasion. Mechanistically, GDPD3 enhances lysophosphatidic acid (LPA) production, which activates the LPAR1–AKT signaling pathway, leading to epithelial–mesenchymal transition (EMT). Inhibition of LPAR1 or AKT effectively reversed LPA-induced EMT, underscoring the critical role of the GDPD3–LPA–LPAR1–AKT axis in driving malignant phenotypes. These findings not only reveal a novel molecular mechanism underlying prostate cancer aggressiveness but also highlight GDPD3 as a promising therapeutic target for future intervention strategies.

## Data Availability

The original contributions presented in the study are included in the article/**Supplementary Material**. Further inquiries can be directed to the corresponding authors.
