# Supplementary figures and images for: Identify GDPD3 as a key regulator of epithelial–mesenchymal transition and prostate adenocarcinoma progression via the LPA/LPAR1/AKT axis: transcriptomic and experimental study
Source: Front Immunol. 2026 Jan 5;16:1637325. doi: 10.3389/fimmu.2025.1637325 (PMC12813044; doi:10.3389/fimmu.2025.1637325)

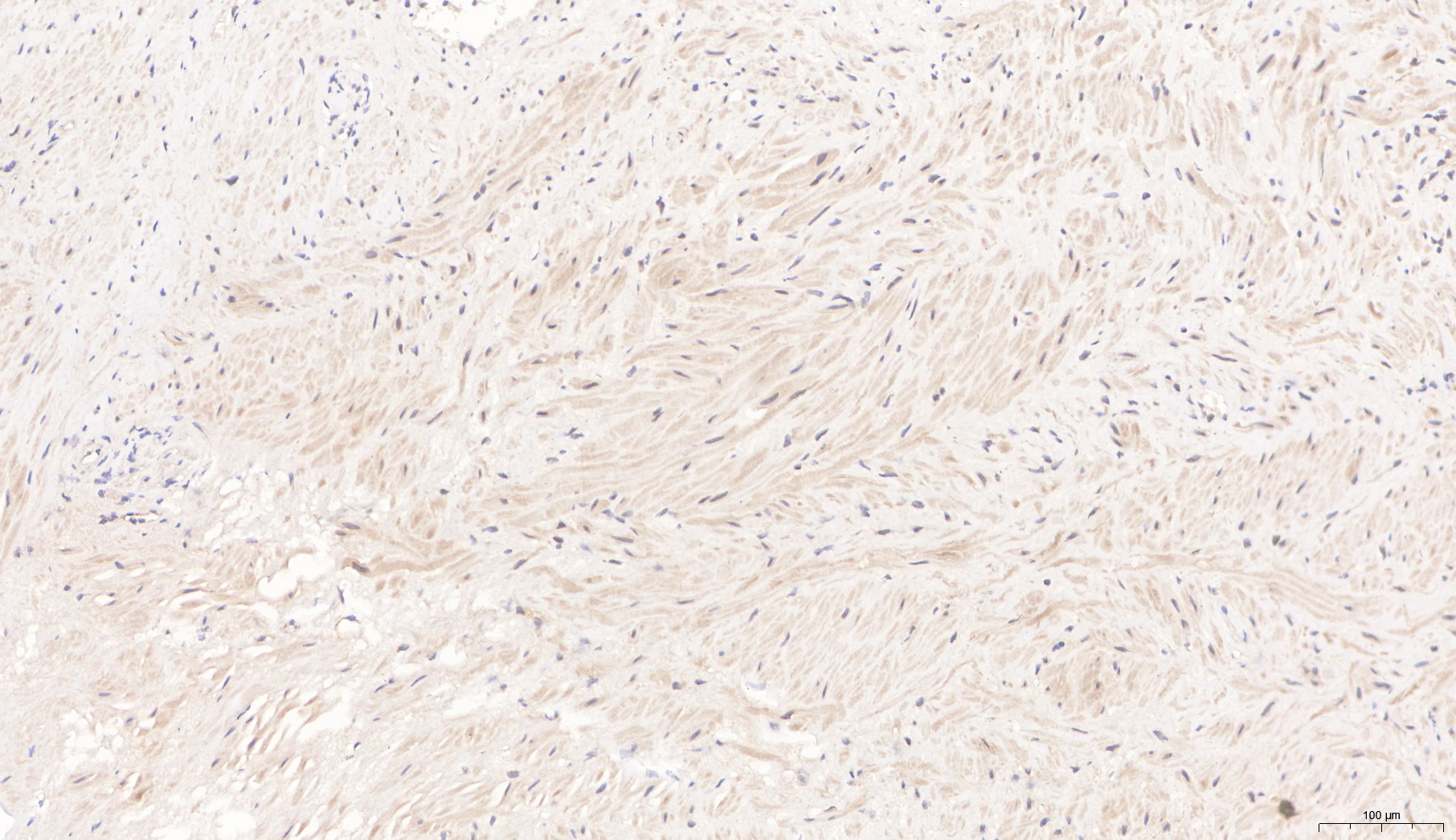

Supplement: Supplementary file 1 [file DataSheet1.zip › Original Images/Fig9-B/1.png]

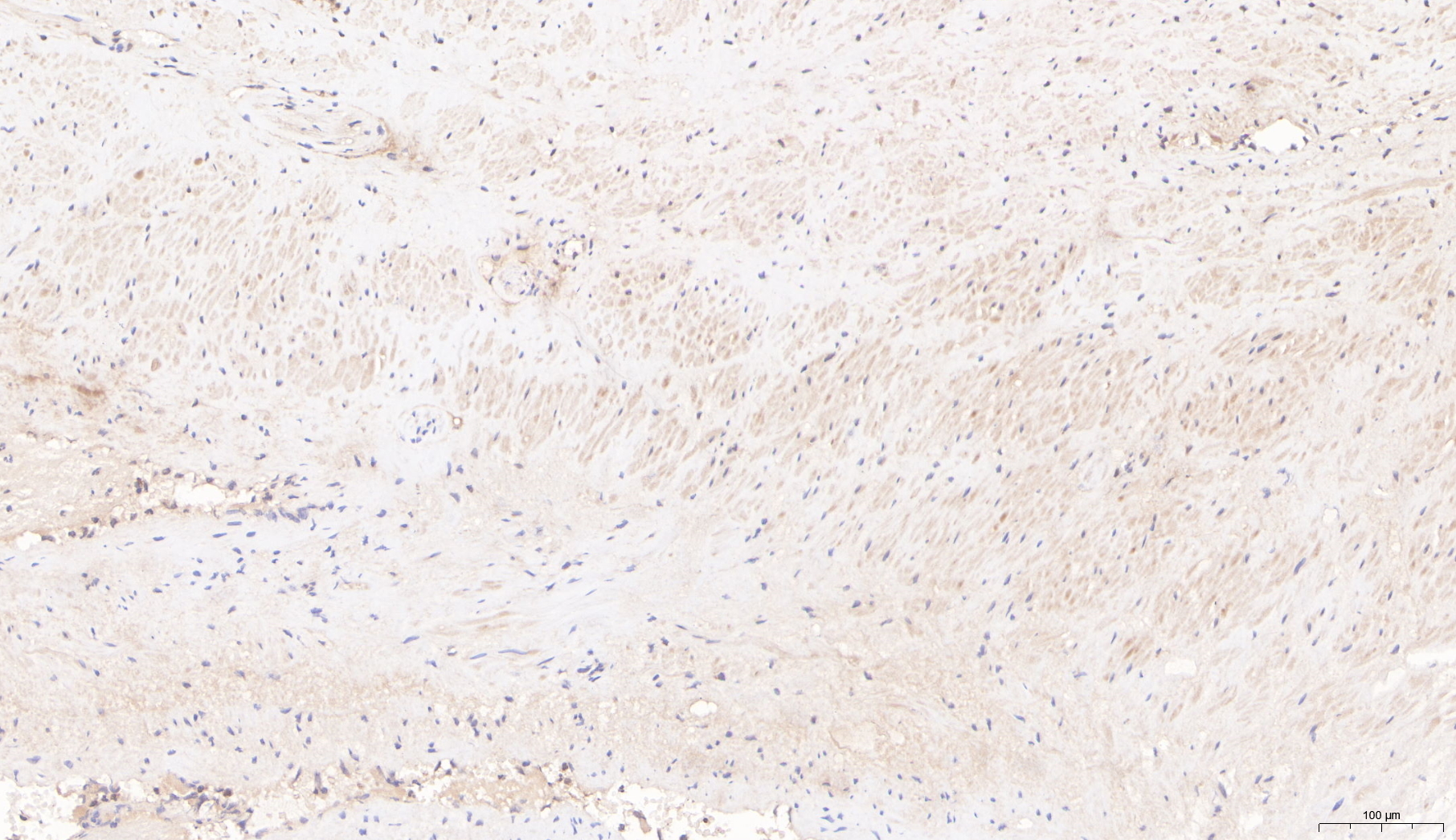

Supplement: Supplementary file 1 [file DataSheet1.zip › Original Images/Fig9-B/2.jpg]

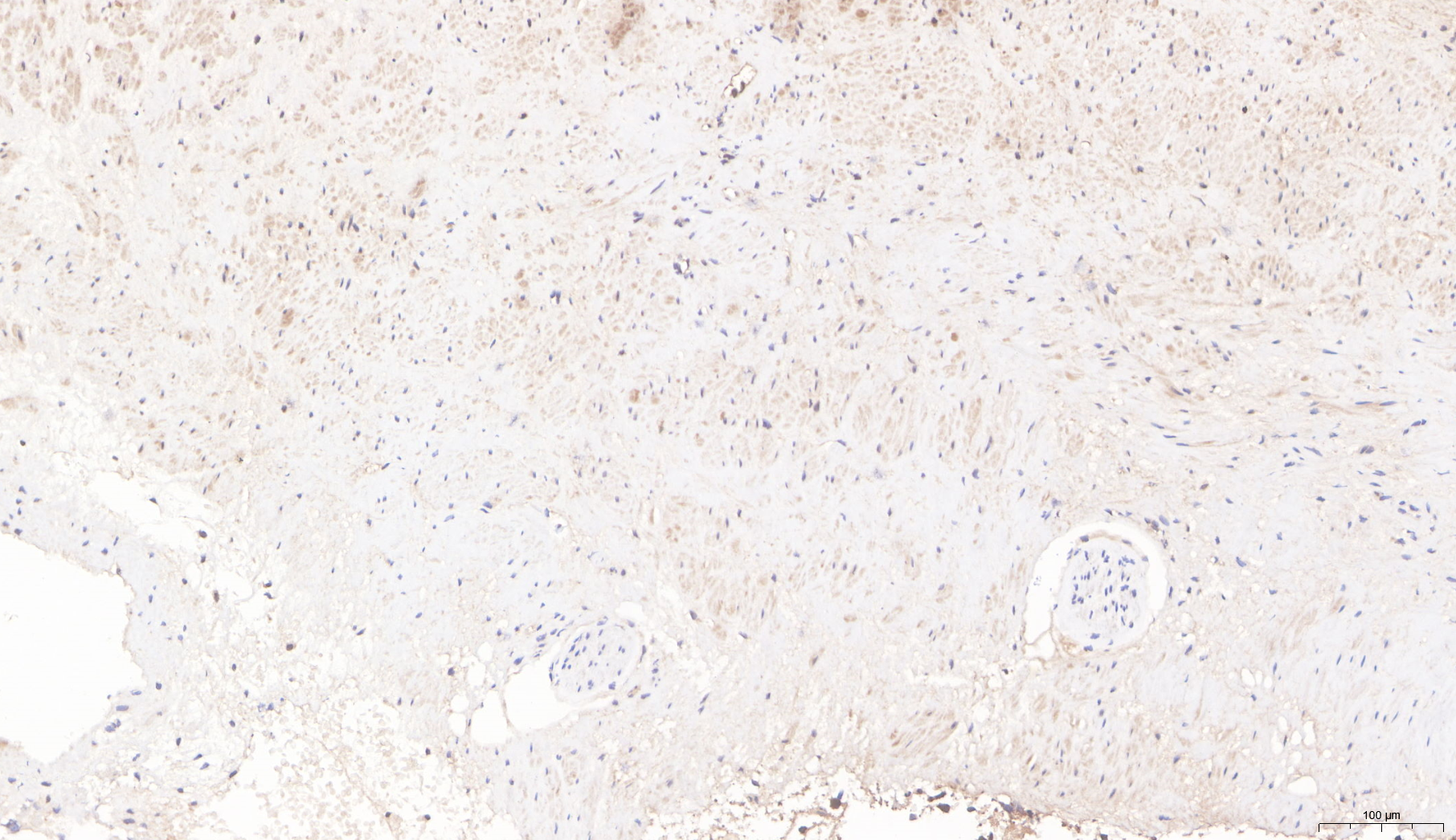

Supplement: Supplementary file 1 [file DataSheet1.zip › Original Images/Fig9-B/3.png]

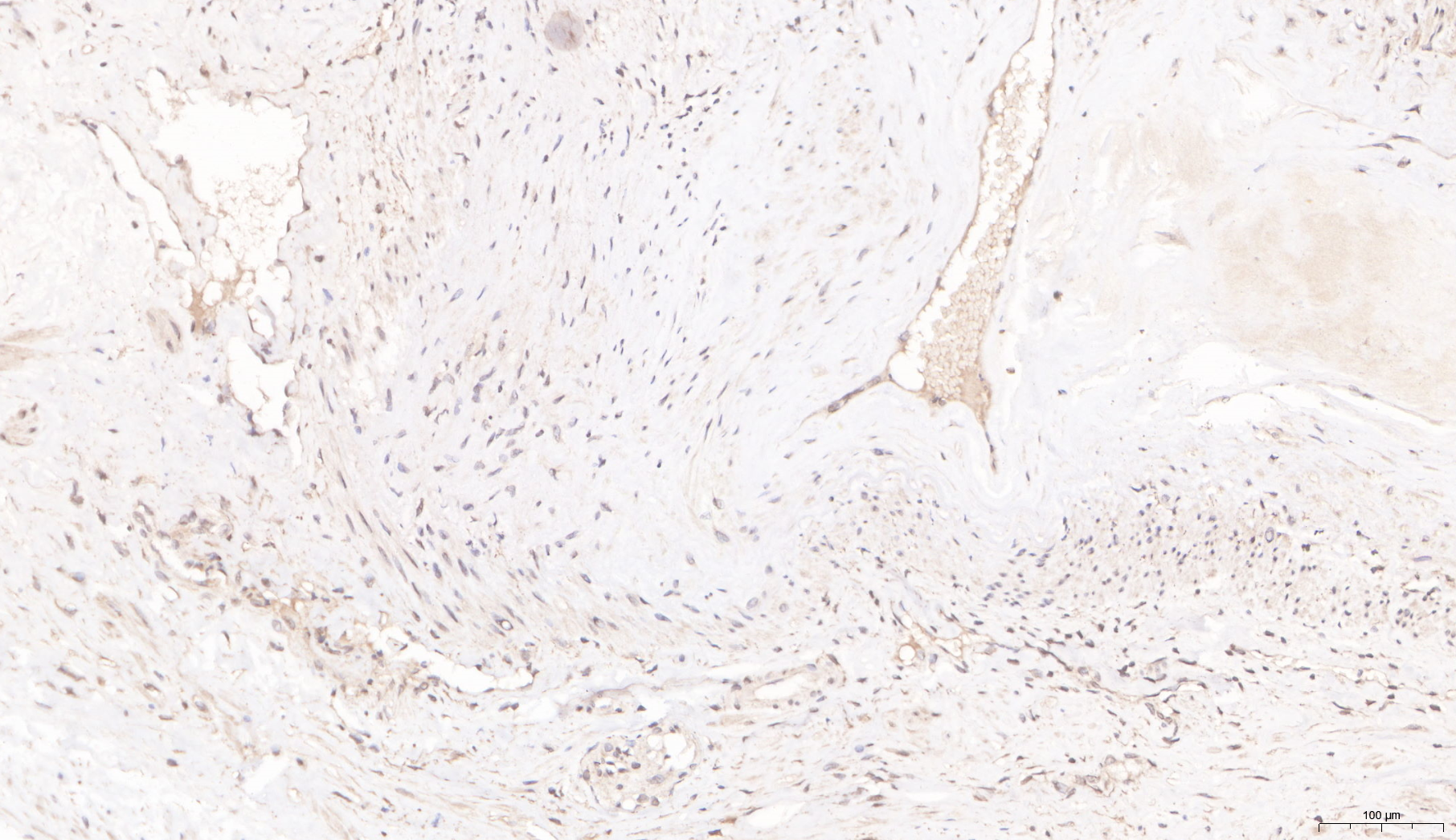

Supplement: Supplementary file 1 [file DataSheet1.zip › Original Images/Fig9-B/4.png]

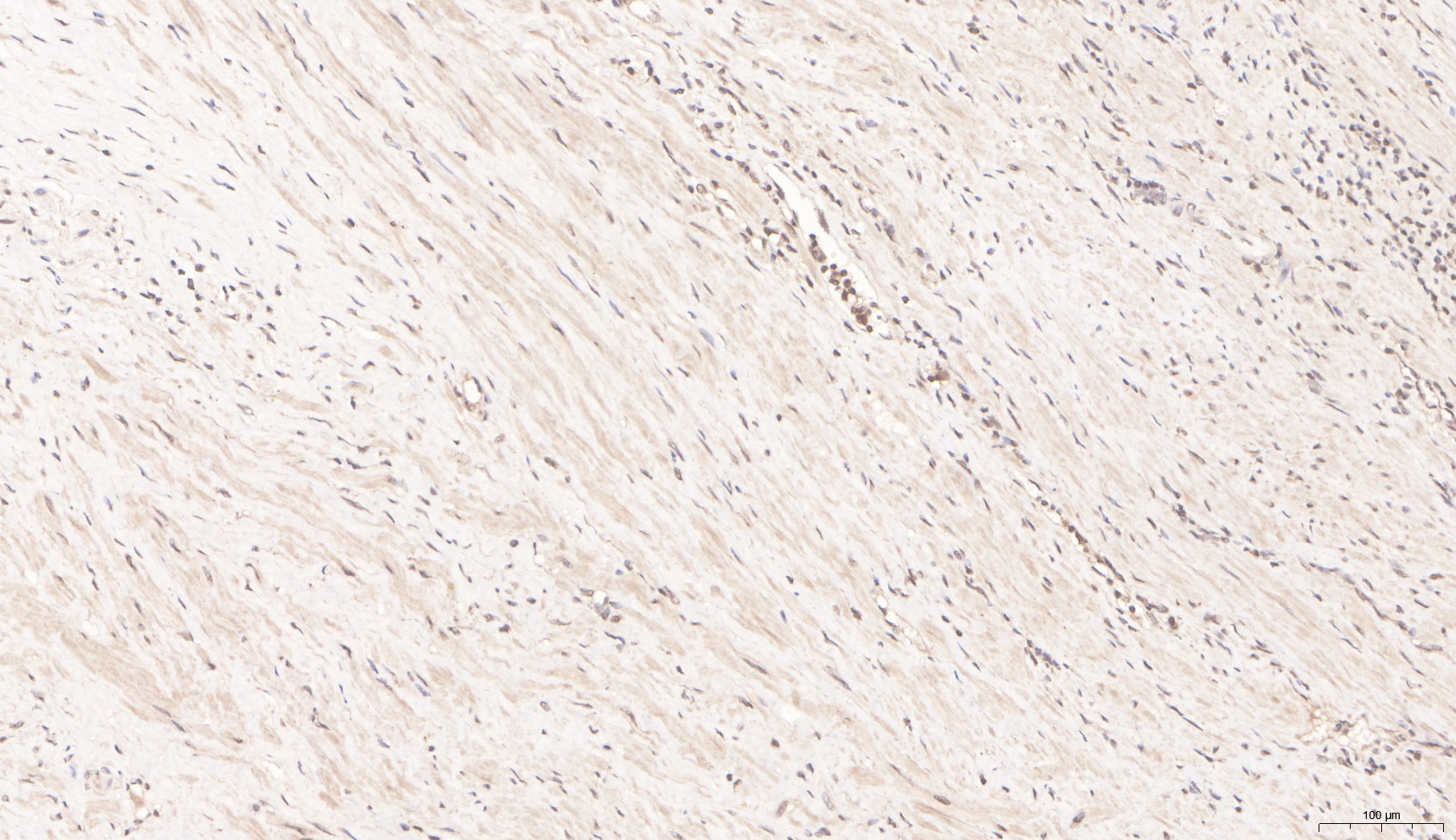

Supplement: Supplementary file 1 [file DataSheet1.zip › Original Images/Fig9-B/5.png]

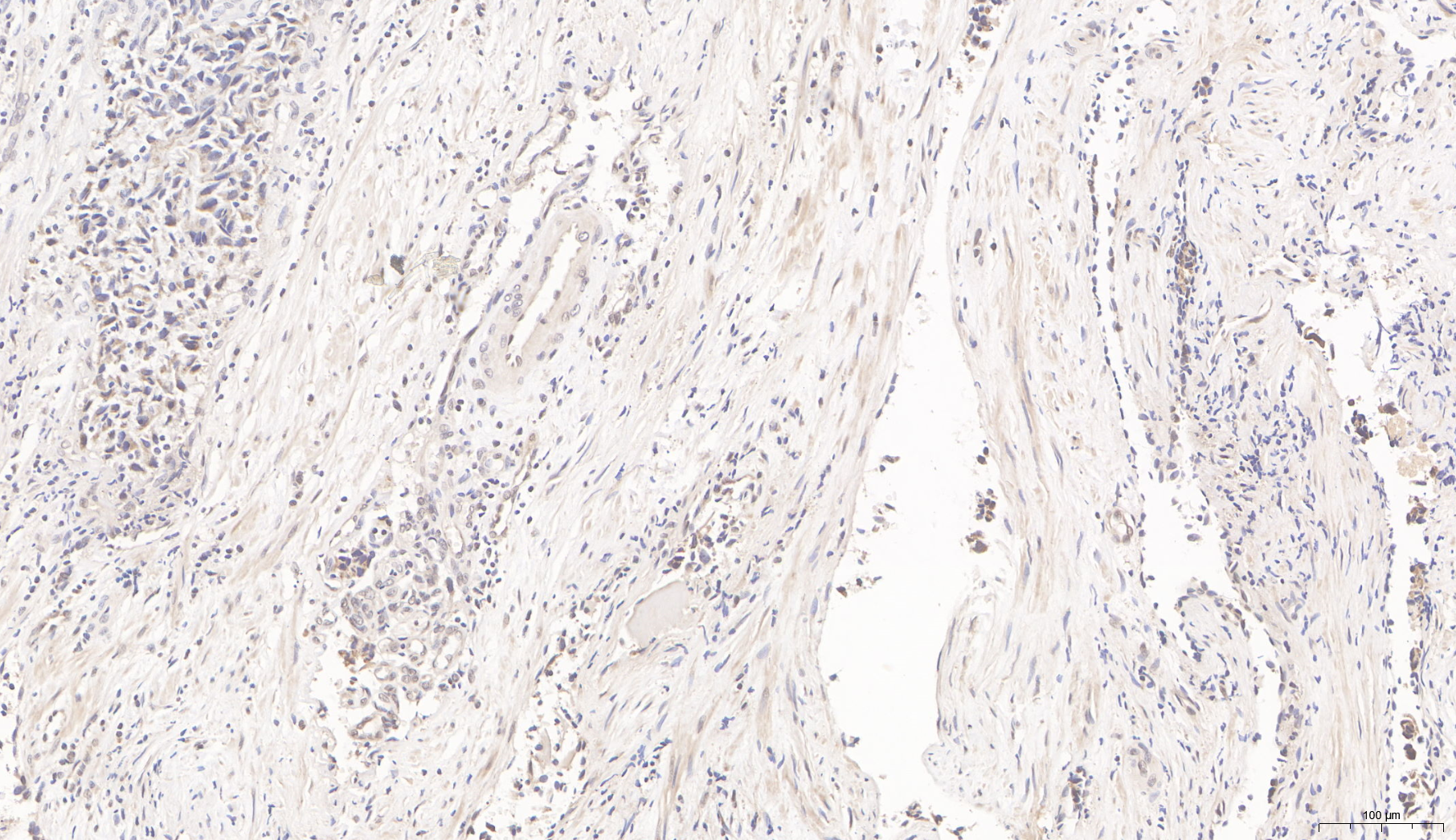

Supplement: Supplementary file 1 [file DataSheet1.zip › Original Images/Fig9-B/6.png]

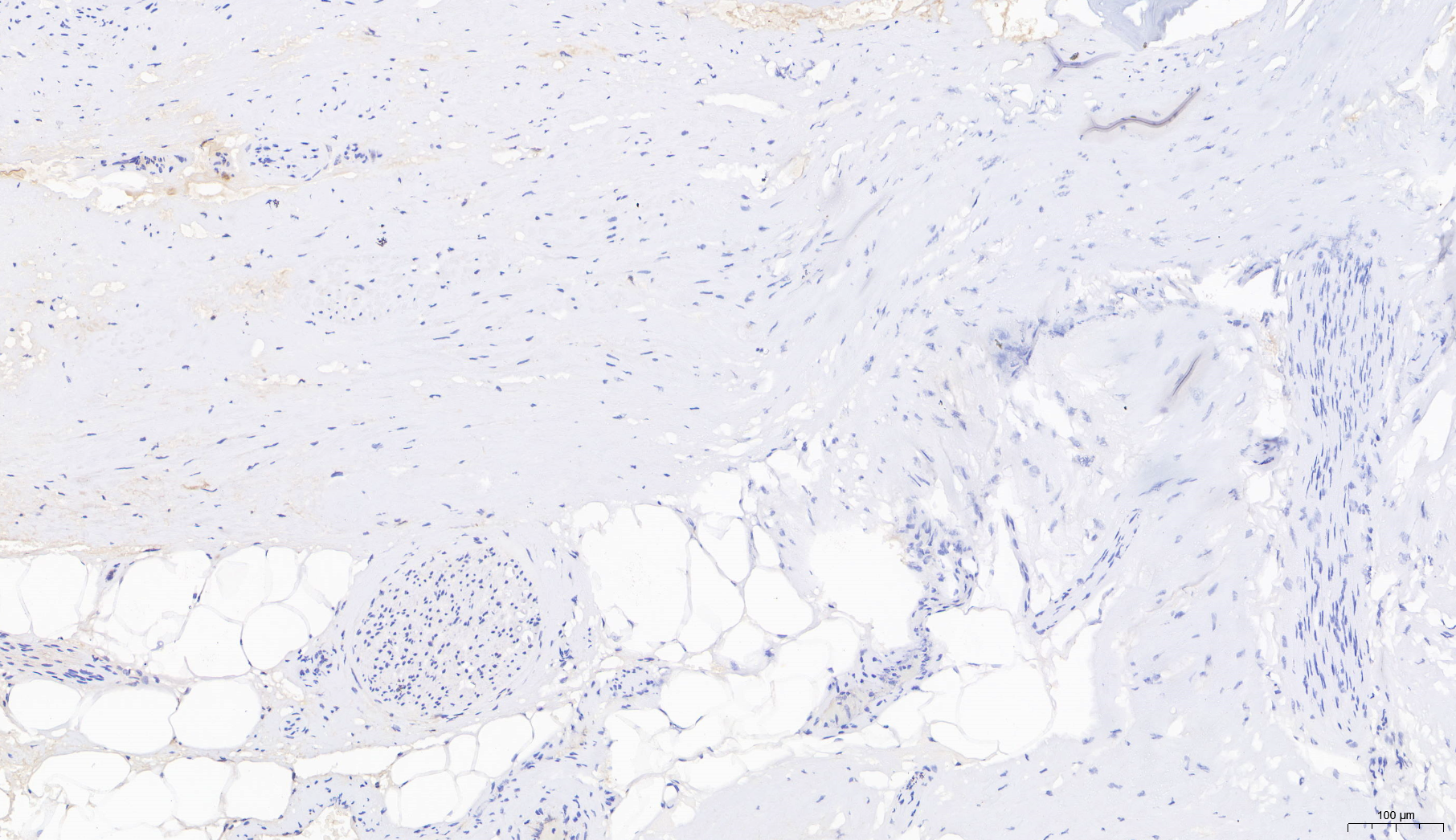

Supplement: Supplementary file 1 [file DataSheet1.zip › Original Images/Fig9-B/7.png]

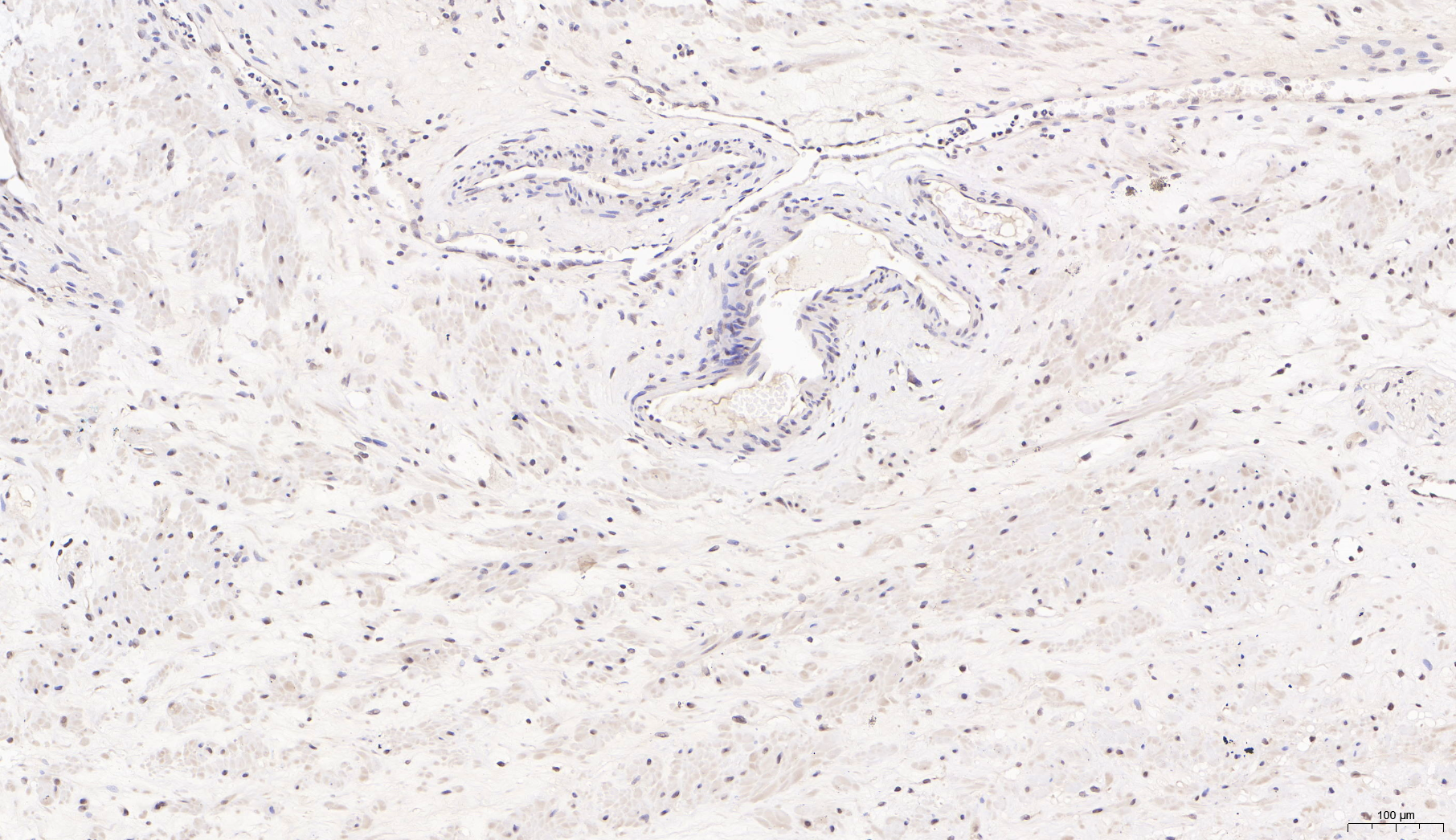

Supplement: Supplementary file 1 [file DataSheet1.zip › Original Images/Fig9-B/8.png]

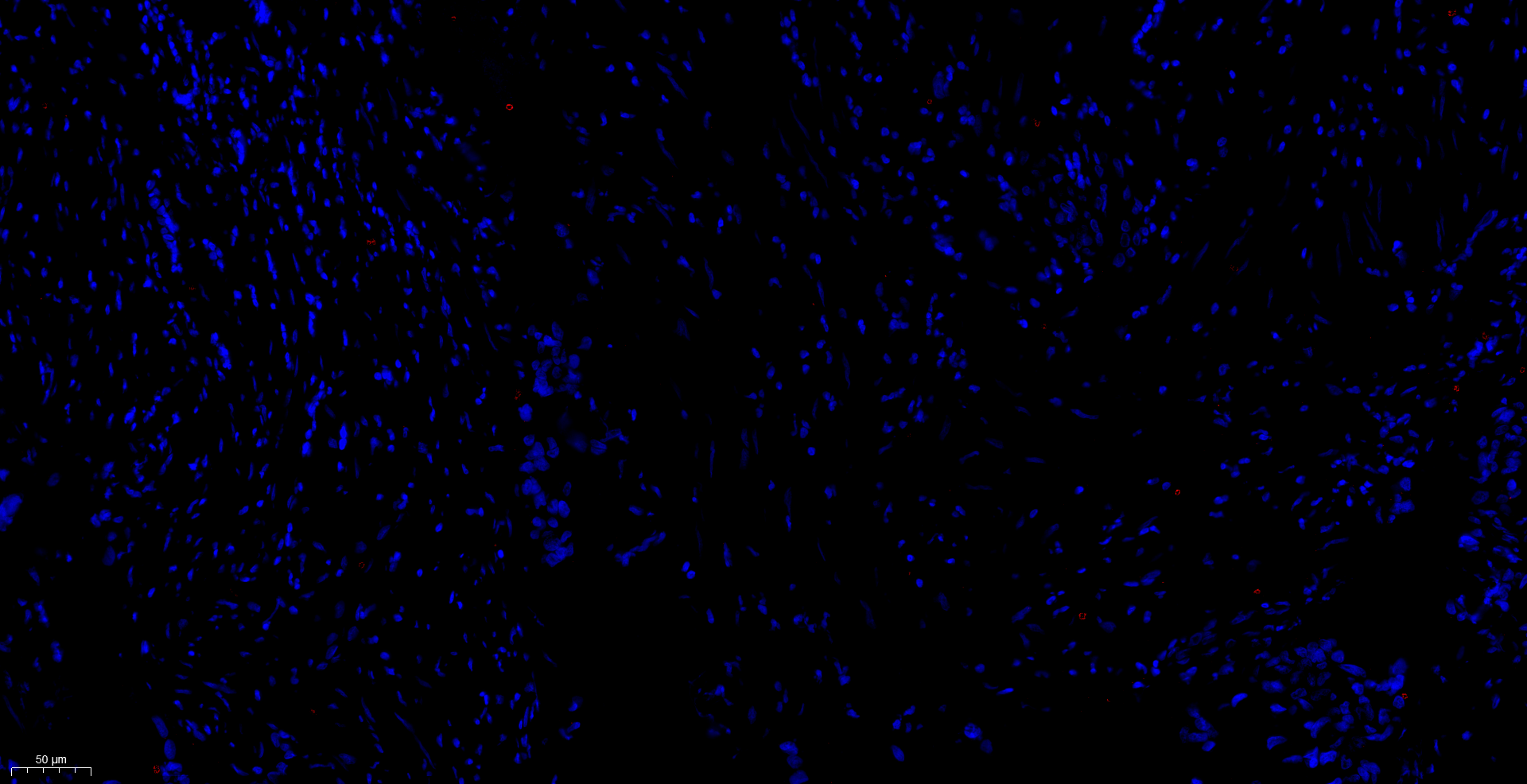

Supplement: Supplementary file 1 [file DataSheet1.zip › Original Images/Fig9-C/2227012 A12 IF PSA红_nor3_20.0x.png]

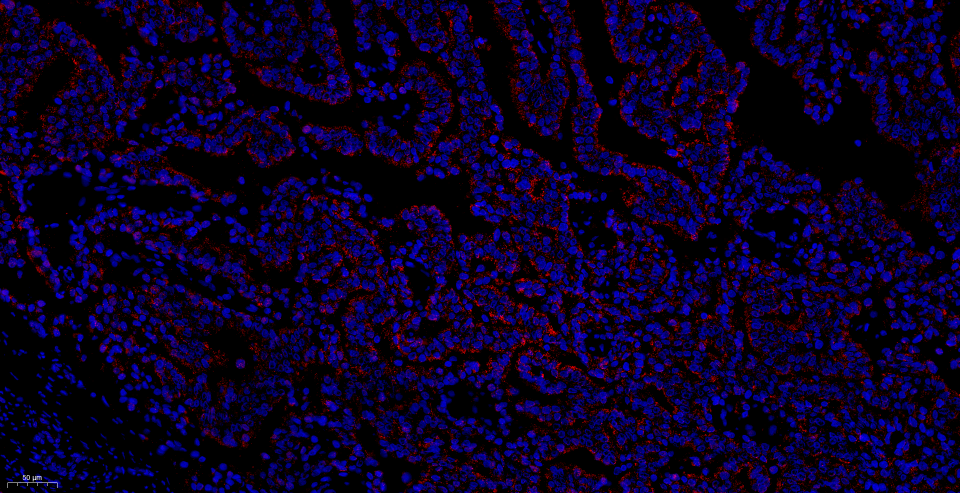

Supplement: Supplementary file 1 [file DataSheet1.zip › Original Images/Fig9-C/2303960 B4 IF GDPD3红_中3_20.0x.png]

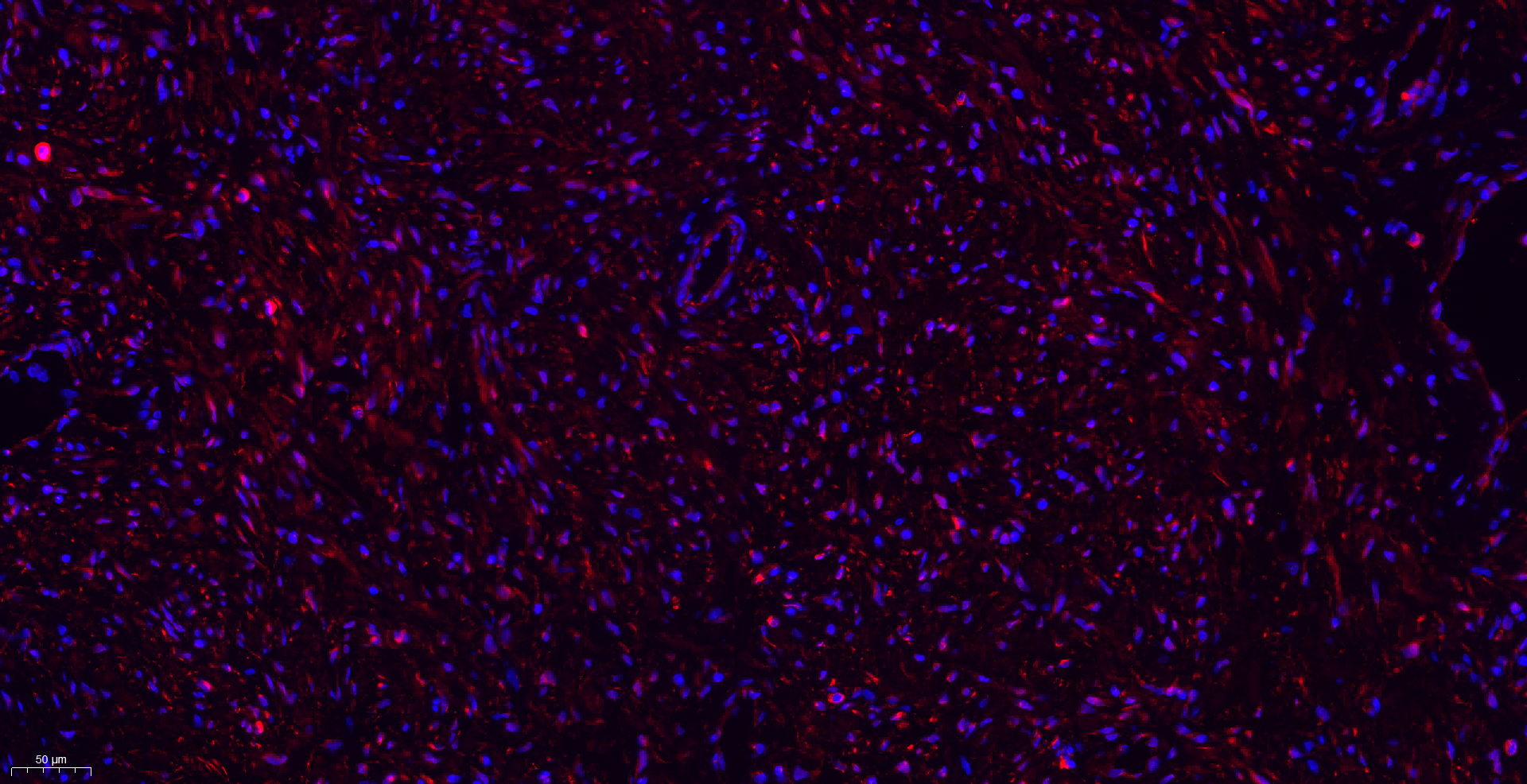

Supplement: Supplementary file 1 [file DataSheet1.zip › Original Images/Fig9-C/2324962 A3 IF GDPD3红_重3_20.0x.png]

Figure 10C

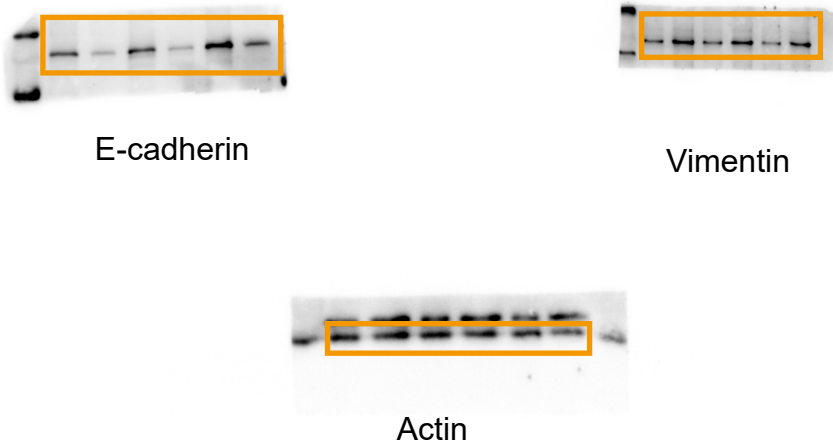

Figure 10D

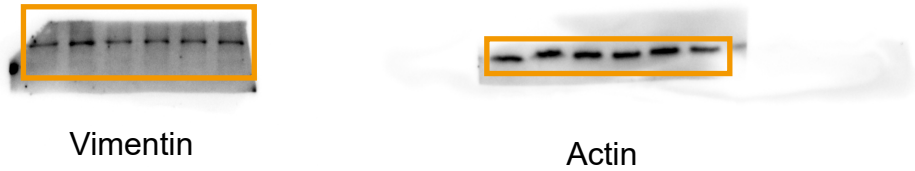

Figure 10E

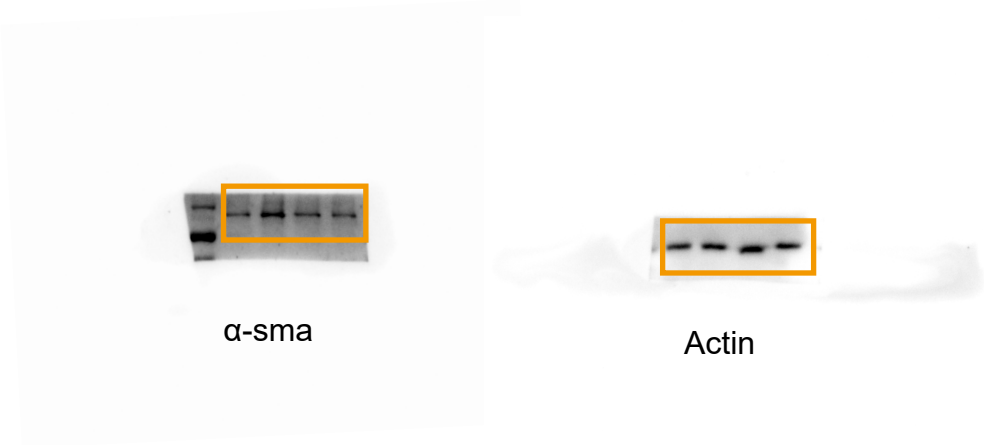

Supplement: Supplementary file 1 [file DataSheet1.zip › Original Images/fig10 WB original gel.pdf]

Figure11 A

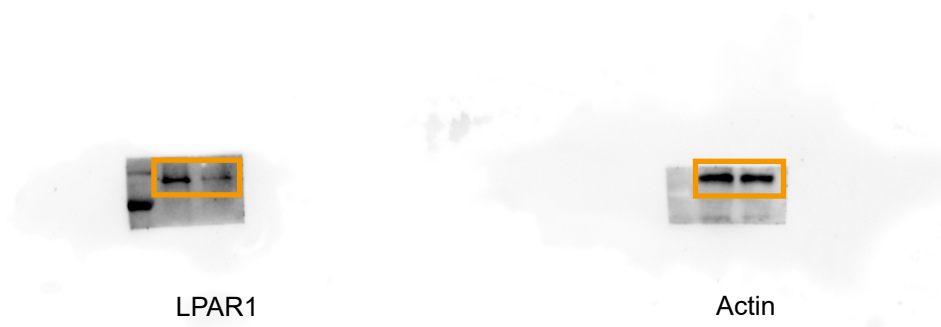

Figure11 C

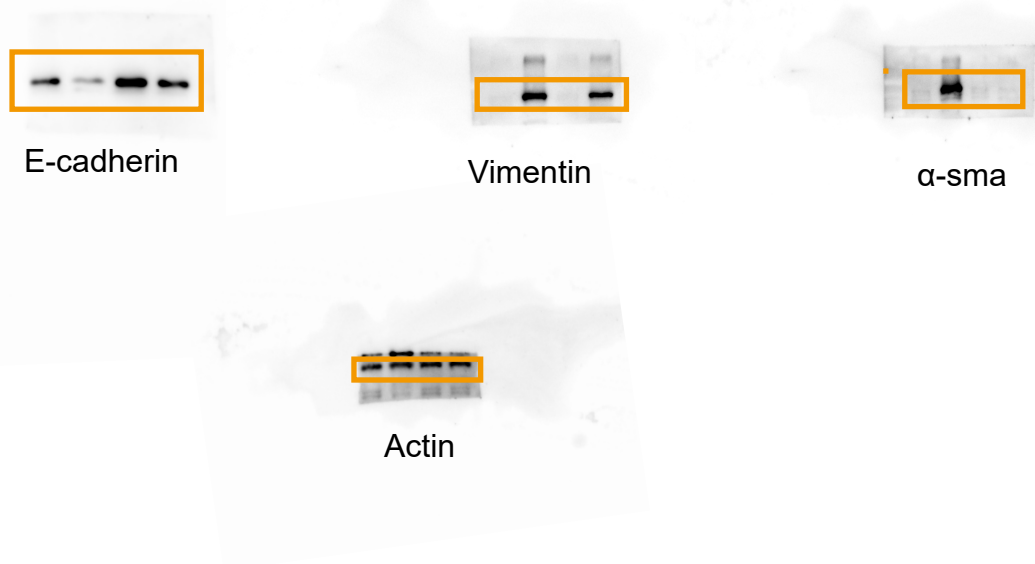

Figure11 E

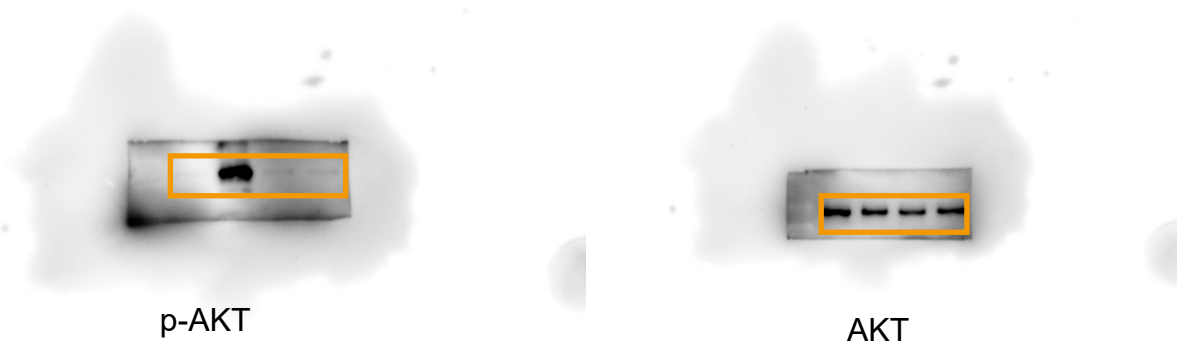

Figure11 G

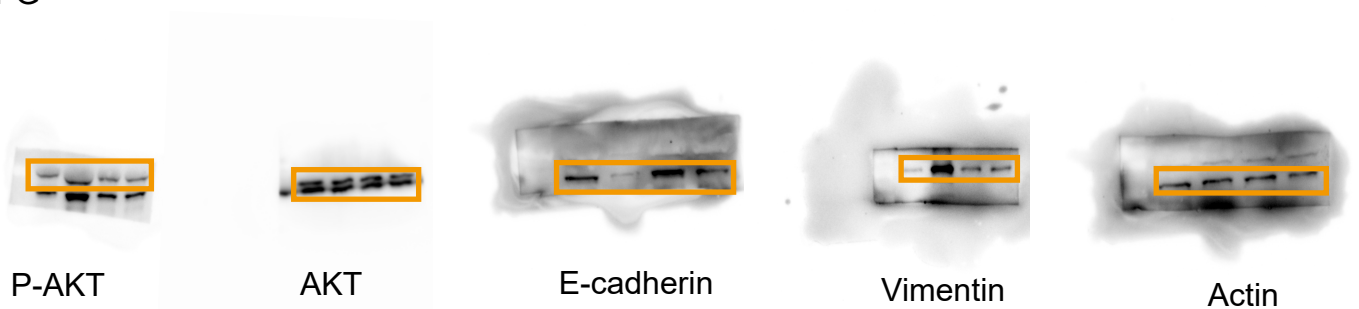

Supplement: Supplementary file 1 [file DataSheet1.zip › Original Images/fig11 WB original gel.pdf]
