# Supplementary material for: Identify GDPD3 as a key regulator of epithelial–mesenchymal transition and prostate adenocarcinoma progression via the LPA/LPAR1/AKT axis: transcriptomic and experimental study
Source: Front Immunol. 2026 Jan 5;16:1637325. doi: 10.3389/fimmu.2025.1637325 (PMC12813044; doi:10.3389/fimmu.2025.1637325)
Supplement: Supplementary file 2 [file DataSheet2.pdf]

Supplementary Material

Supplementary Figures and Legends

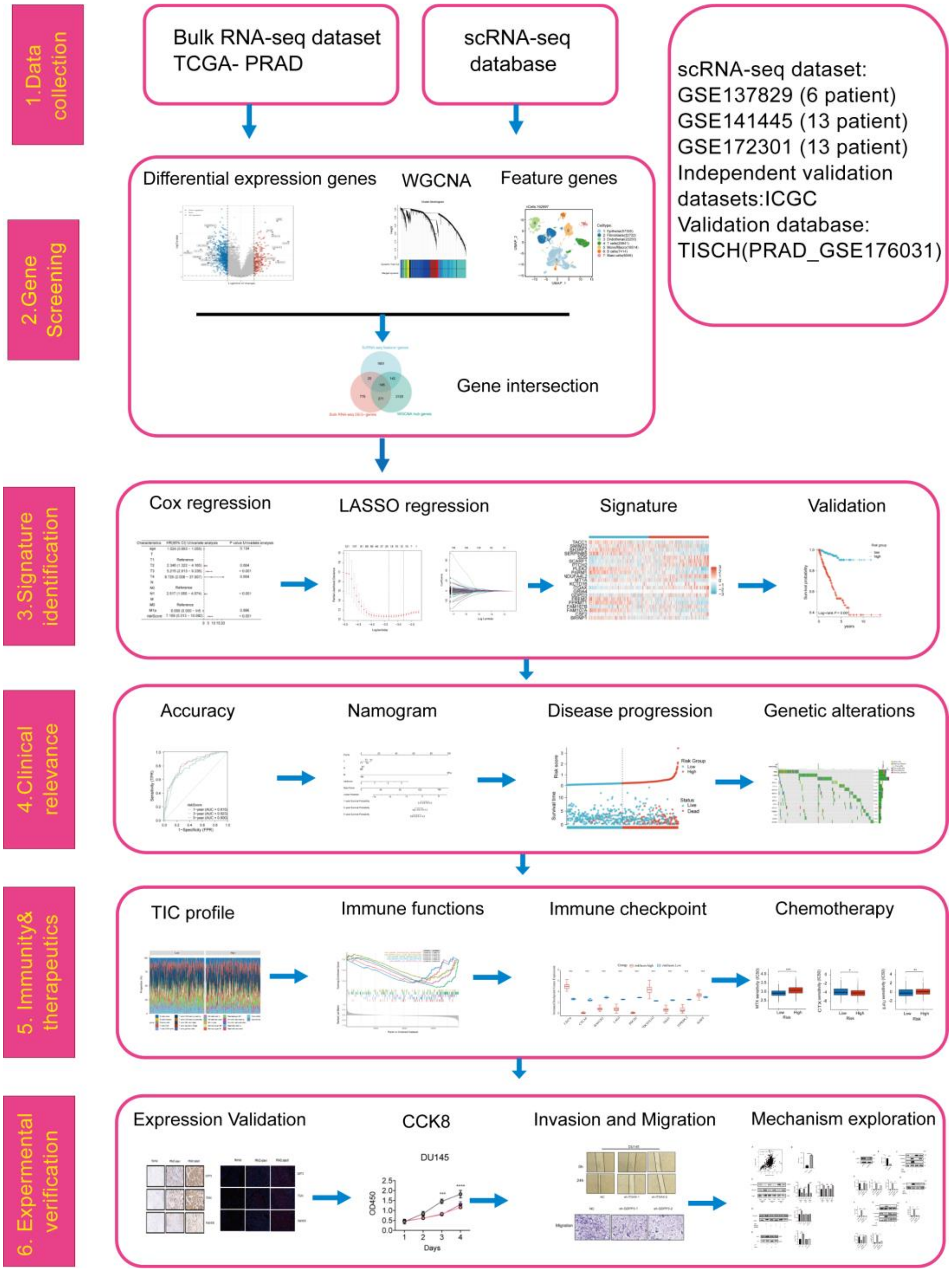

Figure.S1 Flow chart of the present study.

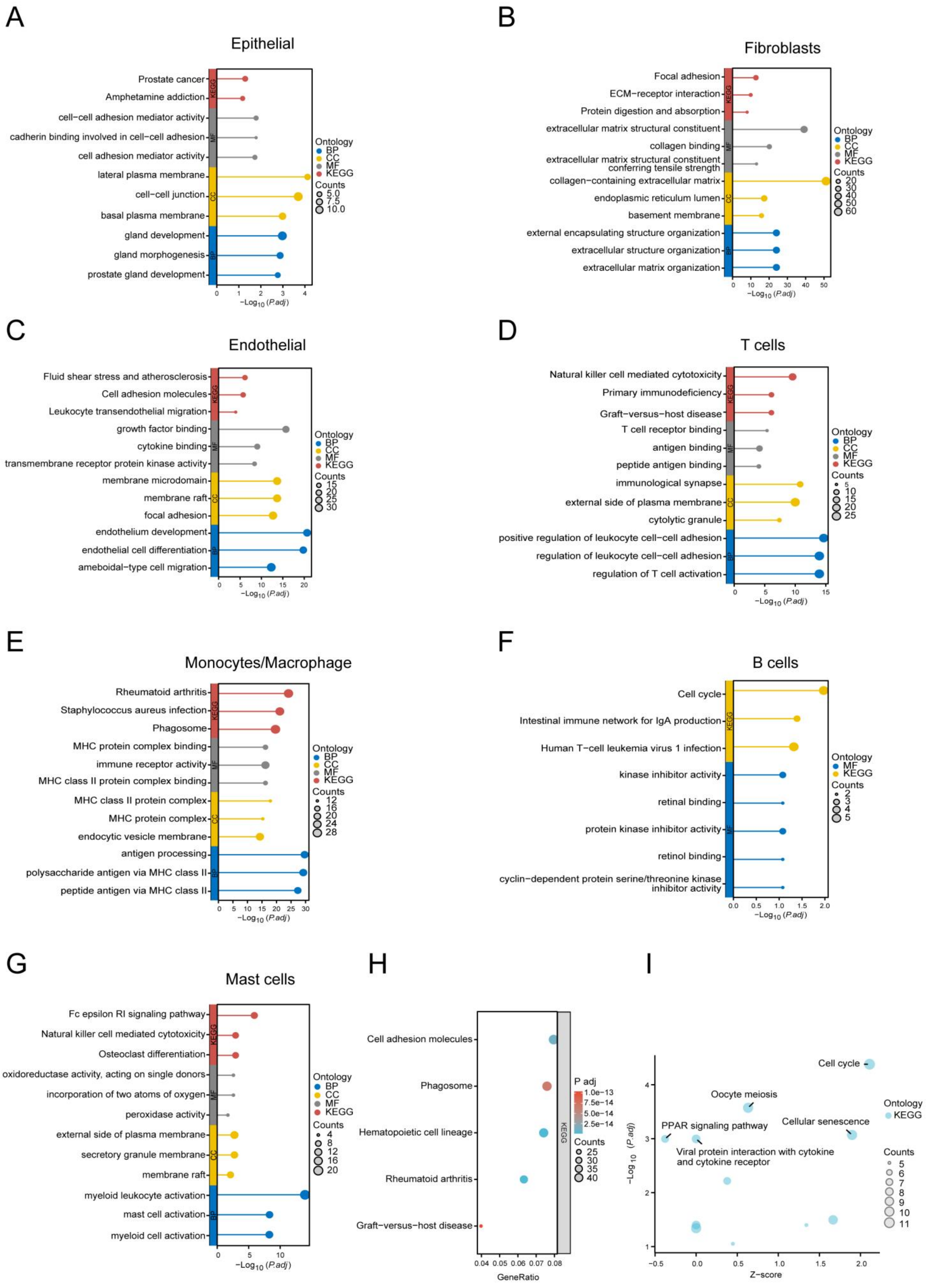
A
Epithelial
Prostate cancer
Amphetamine addiction
cell−cell adhesion mediator activity
cadherin binding involved in cell−cell adhesion
cell adhesion mediator activity
lateral plasma membrane
cell−cell junction
basal plasma membrane
gland development
gland morphogenesis
prostate gland development
KEGG
MF
CC
BP
−Log10 (P.adj)
Ontology
BP
CC
MF
KEGG
Counts
5.0
7.5
10.0
B
Fibroblasts
Focal adhesion
ECM−receptor interaction
Protein digestion and absorption
extracellular matrix structural constituent
collagen binding
extracellular matrix structural constituent conferring tensile strength
collagen−containing extracellular matrix
endoplasmic reticulum lumen
basement membrane
external encapsulating structure organization
extracellular structure organization
extracellular matrix organization
Counts
20
30
40
50
60
C
Endothelial
Fluid shear stress and atherosclerosis
Cell adhesion molecules
Leukocyte transendothelial migration
growth factor binding
cytokine binding
transmembrane receptor protein kinase activity
membrane microdomain
membrane raft
focal adhesion
endothelium development
endothelial cell differentiation
ameboidal−type cell migration
Counts
15
20
25
30
D
T cells
Natural killer cell mediated cytotoxicity
Primary immunodeficiency
Graft−versus−host disease
T cell receptor binding
antigen binding
peptide antigen binding
immunological synapse
external side of plasma membrane
cytolytic granule
positive regulation of leukocyte cell−cell adhesion
regulation of leukocyte cell−cell adhesion
regulation of T cell activation
Counts
5
10
15
20
25
E
Monocytes/Macrophage
Rheumatoid arthritis
Staphylococcus aureus infection
Phagosome
MHC protein complex binding
immune receptor activity
MHC class II protein complex binding
MHC class II protein complex
MHC protein complex
endocytic vesicle membrane
antigen processing
polysaccharide antigen via MHC class II
peptide antigen via MHC class II
Counts
12
16
20
24
28
F
B cells
Cell cycle
Intestinal immune network for IgA production
Human T−cell leukemia virus 1 infection
kinase inhibitor activity
retinal binding
protein kinase inhibitor activity
retinol binding
cyclin−dependent protein serine/threonine kinase inhibitor activity
Ontology
MF
KEGG
Counts
2
3
4
5
G
Mast cells
Fc epsilon RI signaling pathway
Natural killer cell mediated cytotoxicity
Osteoclast differentiation
oxidoreductase activity, acting on single donors
incorporation of two atoms of oxygen
peroxidase activity
external side of plasma membrane
secretory granule membrane
membrane raft
myeloid leukocyte activation
mast cell activation
myeloid cell activation
Counts
4
8
12
16
20
H
Cell adhesion molecules
Phagosome
Hematopoietic cell lineage
Rheumatoid arthritis
Graft−versus−host disease
KEGG
P adj
1.0e−13
7.5e−14
5.0e−14
2.5e−14
Counts
25
30
35
40
GeneRatio
I
Cell cycle
Oocyte meiosis
PPAR signaling pathway
Cellular senescence
Viral protein interaction with cytokine and cytokine receptor
Ontology
KEGG
Counts
5
6
7
8
9
10
11
−Log 10 (P.adj)
Z−score

Figure. S2 Pathway Enrichment Across Cell Types and Bulk Transcriptomes

(A-G). KEGG and GO enrichment analysis of fibroblasts, endothelial cells, epithelial cells, monocytes/macrophages, B cells, T cells, and mast cells. (H). scRNA-seq Top 5 pathways. (I). TCGA-PRAD Top 5 pathways.

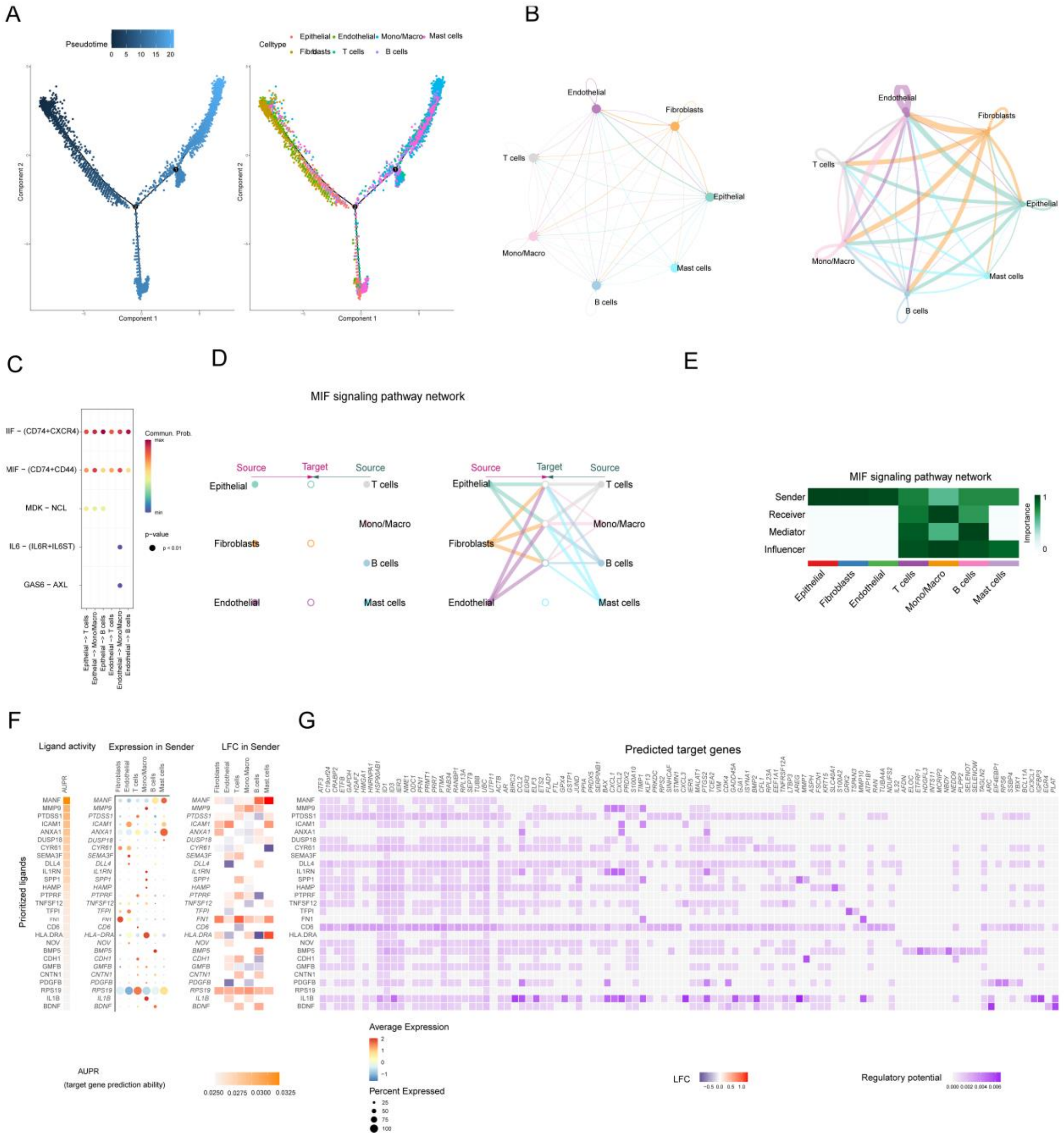

Figure. S3 Investigation of PRAD cell trajectory and cell-cell communication with diverse differentiation patterns.

(A) . Analysis of the trajectory of PRAD cells indicated two primary branches: one dominated by epithelial cells, endothelial cells, and fibroblasts, while the other was dominated by immune cells **(B).** Number and strength of interactions among different cell types **(C).** Bubble diagram illustrating cell

communication signaling route strength. **(D).** MIF signaling pathway network. **(E).** MIF senders include epithelial, endothelial, and fibroblasts. **(F-G).** NicheNet analysis of ligand-receptor interactions.

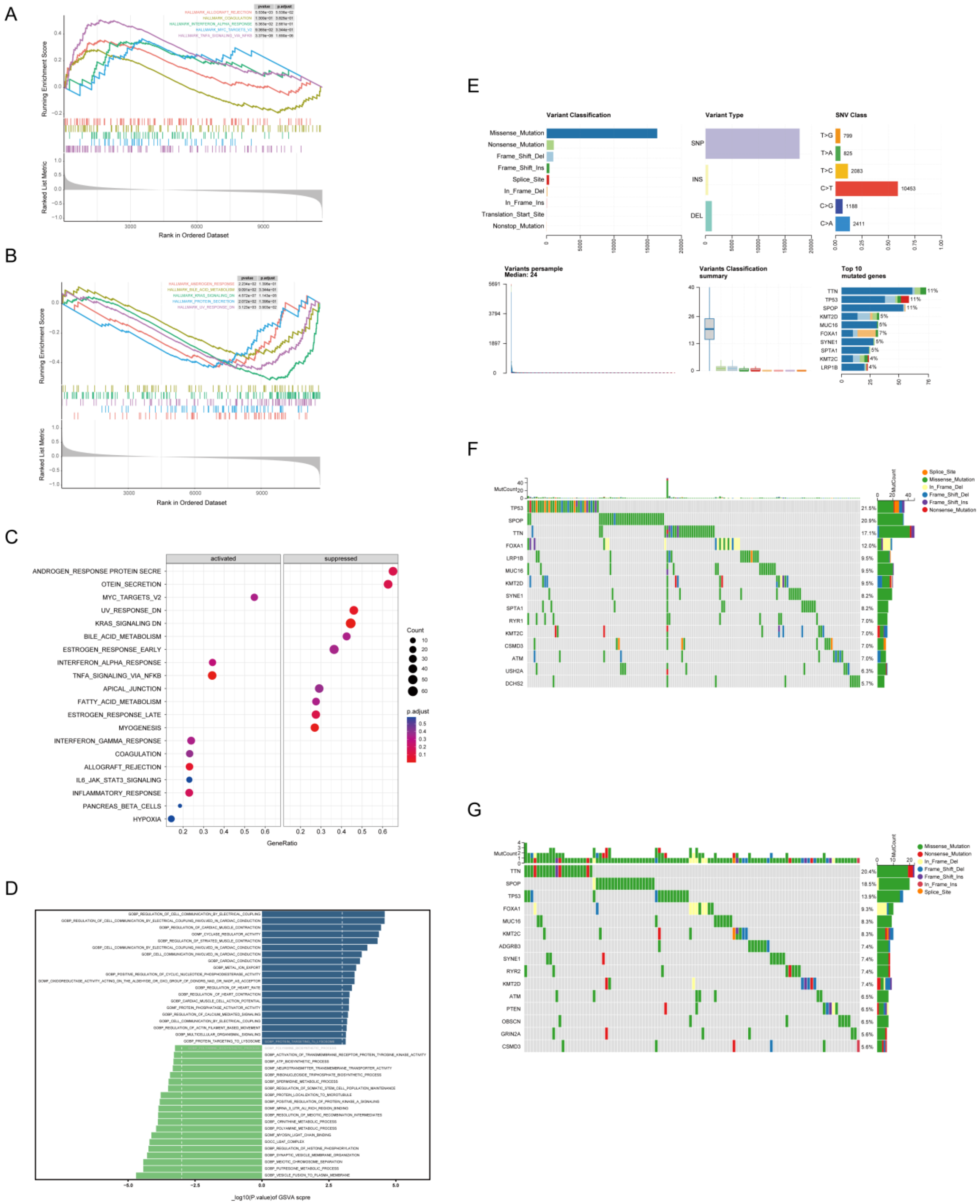
A
Running Enrichment Score
Ranked List Metric
Rank in Ordered Dataset
B
Running Enrichment Score
Ranked List Metric
Rank in Ordered Dataset
C
activated
suppressed
ANDROGEN_RESPONSE PROTEIN SECRE
OTEIN_SECRETION
MYC_TARGETS_V2
UV_RESPONSE_DN
KRAS_SIGNALING DN
BILE_ACID_METABOLISM
ESTROGEN_RESPONSE_EARLY
INTERFERON_ALPHA_RESPONSE
TNFA_SIGNALING_VIA_NFKB
APICAL_JUNCTION
FATTY_ACID_METABOLISM
ESTROGEN_RESPONSE_LATE
MYOGENESIS
INTERFERON_GAMMA_RESPONSE
COAGULATION
ALLOGRAFT_REJECTION
IL6_JAK_STAT3_SIGNALING
INFLAMMATORY_RESPONSE
PANCREAS_BETA_CELLS
HYPOXIA
Count
p.adjust
GeneRatio
D
_log10(P.value)of GSVA scpre
E
Variant Classification
Missense_Mutation
Nonsense_Mutation
Frame_Shift_Del
Frame_Shift_Ins
Splice_Site
In_Frame_Del
In_Frame_Ins
Translation_Start_Site
Nonstop_Mutation
Variant Type
SNP
INS
DEL
SNV Class
T>G 799
T>A 825
T>C 2083
C>T 10453
C>G 1188
C>A 2411
Variants persample
Median: 24
Variants Classification summary
Top 10 mutated genes
TTN 11%
TP53 11%
SPOP 11%
KMT2D 5%
MUC16 5%
FOXA1 7%
SYNE1 5%
SPTA1 5%
KMT2C 4%
LRP1B 4%
F
MutCount
TP53 21.5%
SPOP 20.9%
TTN 17.1%
FOXA1 12.0%
LRP1B 9.5%
MUC16 9.5%
KMT2D 9.5%
SYNE1 8.2%
SPTA1 8.2%
RYR1 7.0%
KMT2C 7.0%
CSMD3 7.0%
ATM 7.0%
USH2A 6.3%
DCHS2 5.7%
Splice_Site
Missense_Mutation
In_Frame_Del
Frame_Shift_Del
Frame_Shift_Ins
Nonsense_Mutation
G
MutCount
TTN 20.4%
SPOP 18.5%
TP53 13.9%
FOXA1 9.3%
MUC16 8.3%
KMT2C 8.3%
ADGRB3 7.4%
SYNE1 7.4%
RYR2 7.4%
KMT2D 7.4%
ATM 6.5%
PTEN 6.5%
OBSCN 6.5%
GRIN2A 5.6%
CSMD3 5.6%
Missense_Mutation
Nonsense_Mutation
In_Frame_Del
Frame_Shift_Del
Frame_Shift_Ins
In_Frame_Ins
Splice_Site

Figure. S4 Biological differences between high- and low-risk groups.

(A, B). GSEA of GO and KEGG between high- and low-risk groups. (C). Visualization of GSEA results in a bubble plot. (D). GSVA performed on all genes within the high- and low-risk groups. (E). An overview of the mutation landscape for TCGA-PRAD patients. (F). Prediction of the tumor mutational burden (TMB) in the high - risk groups using the risk model. (G). Prediction of the tumor mutational burden (TMB) in the low - risk groups using the risk model.

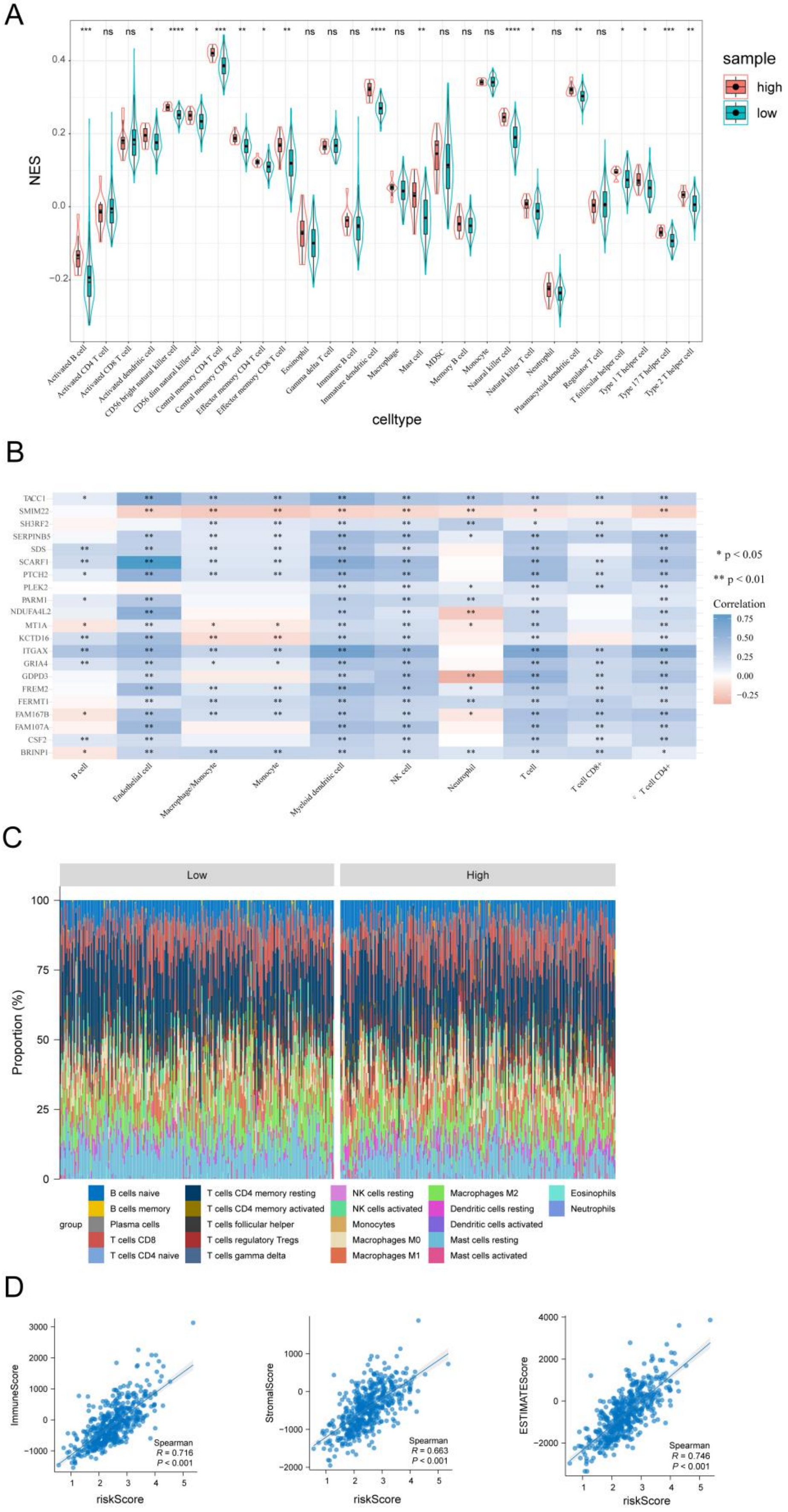
A
sample
high
low
NES
celltype
Activated B cell
Activated CD4 T cell
Activated CD8 T cell
Activated dendritic cell
CD56 bright natural killer cell
CD56 dim natural killer cell
Central memory CD4 T cell
Central memory CD8 T cell
Effector memory CD4 T cell
Effector memory CD8 T cell
Eosinophil
Gamma delta T cell
Immature B cell
Immature dendritic cell
Macrophage
Mast cell
MDSC
Memory B cell
Monocyte
Natural killer cell
Natural killer T cell
Neutrophil
Plasmacytoid dendritic cell
Regulator T cell
T follicular helper cell
Type 1 T helper cell
Type 17 T helper cell
Type 2 T helper cell
B
TACC1
SMIM22
SH3RF2
SERPINB5
SDS
SCARF1
PTCH2
PLEK2
PARM1
NDUFA4L2
MT1A
KCTD16
ITGAX
GRIA4
GDPD3
FREM2
FERMT1
FAM167B
FAM107A
CSF2
BRINP1
B cell
Endothelial cell
Macrophage/Monocyte
Monocyte
Myeloid dendritic cell
NK cell
Neutrophil
T cell
T cell CD8+
T cell CD4+
* p < 0.05
** p < 0.01
Correlation
C
Low
High
Proportion (%)
group
B cells naive
B cells memory
Plasma cells
T cells CD8
T cells CD4 naive
T cells CD4 memory resting
T cells CD4 memory activated
T cells follicular helper
T cells regulatory Tregs
T cells gamma delta
NK cells resting
NK cells activated
Monocytes
Macrophages M0
Macrophages M1
Macrophages M2
Dendritic cells resting
Dendritic cells activated
Mast cells resting
Mast cells activated
Eosinophils
Neutrophils
D
ImmuneScore
riskScore
Spearman
R = 0.716
P < 0.001
StromalScore
riskScore
Spearman
R = 0.663
P < 0.001
ESTIMATEScore
riskScore
Spearman
R = 0.746
P < 0.001

Figure. S5 Immune microenvironment characteristics differ between PRAD risk groups.

(A) . The violin plot illustrating the comparison of ssGSEA scores for 28 immune cells between high and low-risk categories. (B). The link between immune cells and 21 model genes. (C). Tumor-associated immune cell infiltration in high and low riskScore groups. (D). The ESTIMATE algorithm assesses the relationship between riskScore and stromal and immune cell scores.

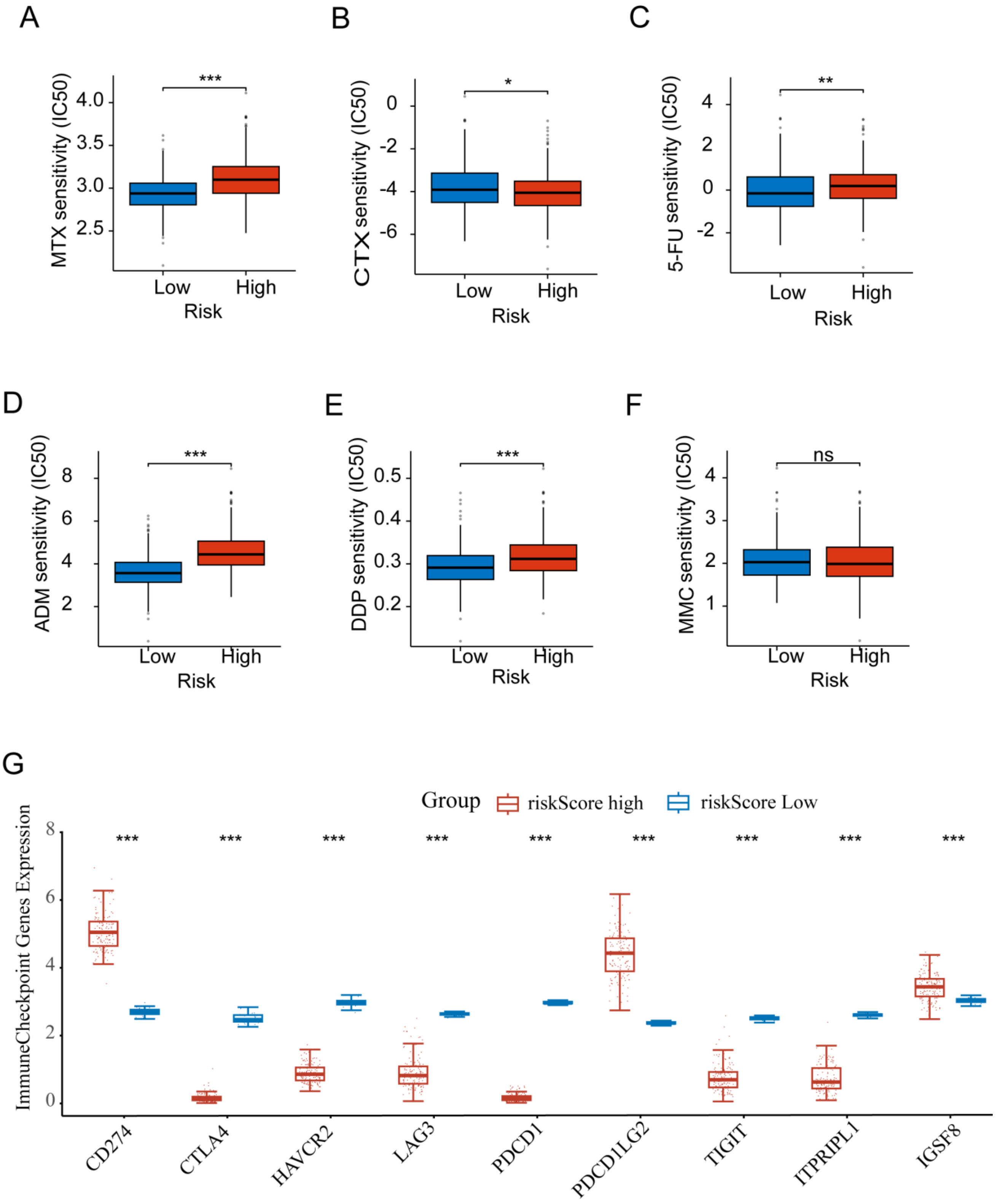

Figure. S6 Chemotherapy sensitivity and immune checkpoint profiles in PRAD risk groups.

(A-F). Determine the IC50 in the two risk groups to evaluate drug sensitivity in a cohort of 420 patients. (G). Assess the immune checkpoints in the two risk groups.

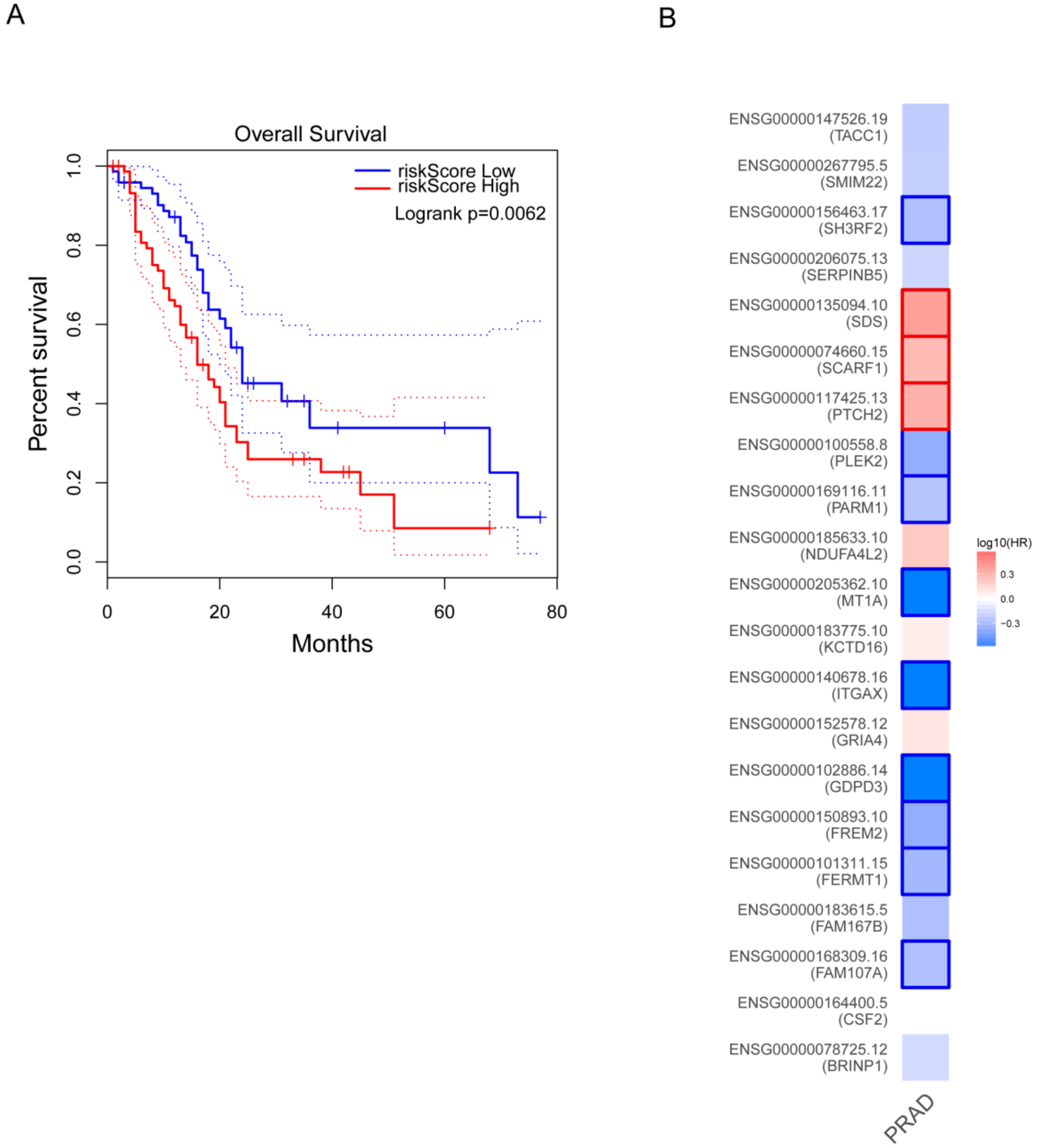

Figure. S7 Validate the robustness of the riskScore using the ICGC database.

(A) **.** The KM survival curve was plotted for high- and low-risk groups. **(B).** The correlation between the model genes and the prognosis of PRAD was evaluated.

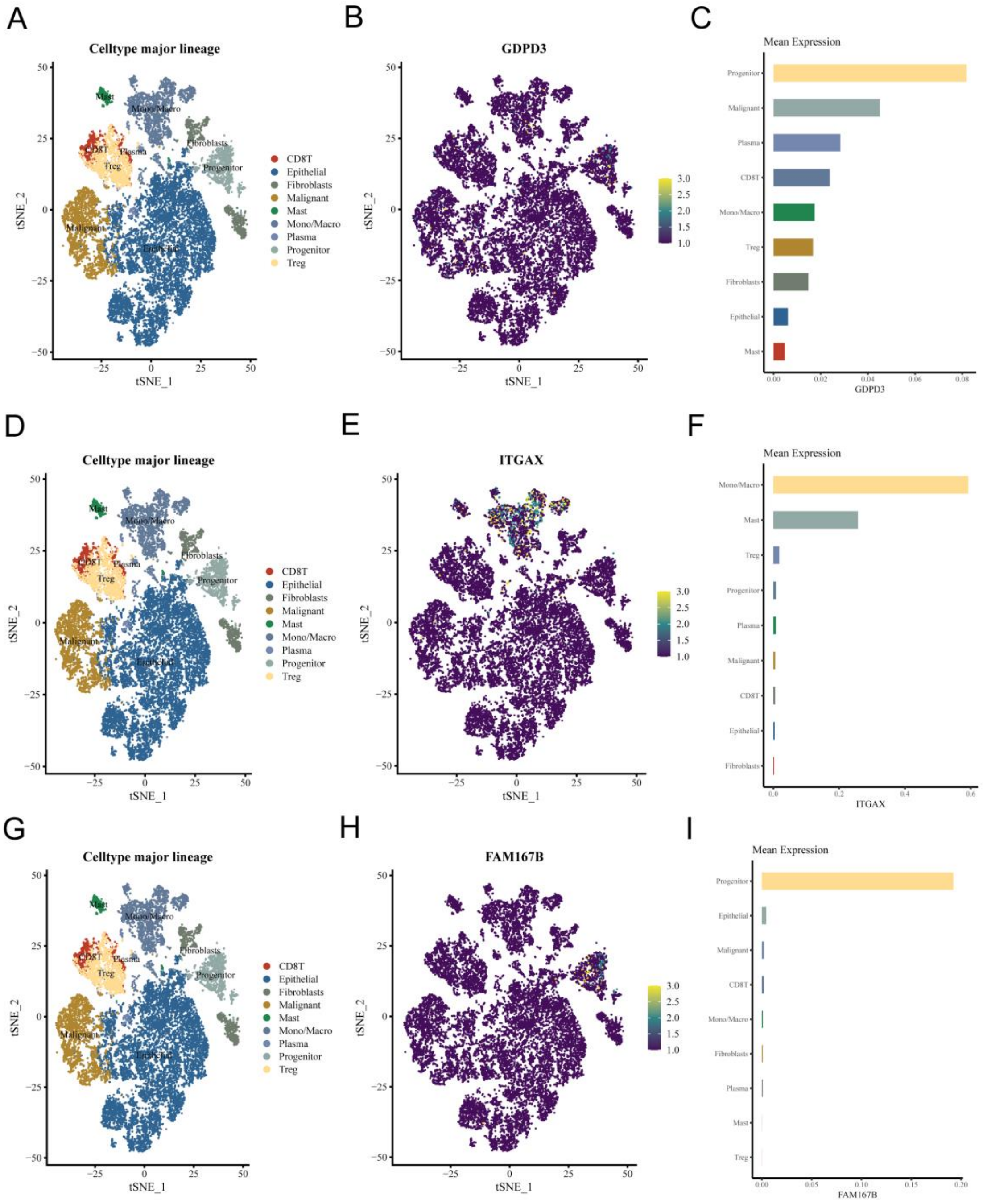

Figure. S8 The expression distribution of risk genes.

(A-C). Display the expression distribution of GDPD3 through single-cell t-SNE. **(D-F).** Display the expression distribution of ITGAX through single-cell t-SNE. **(G-I).** Display the expression distribution of FAM67B through single-cell t-SNE**.**

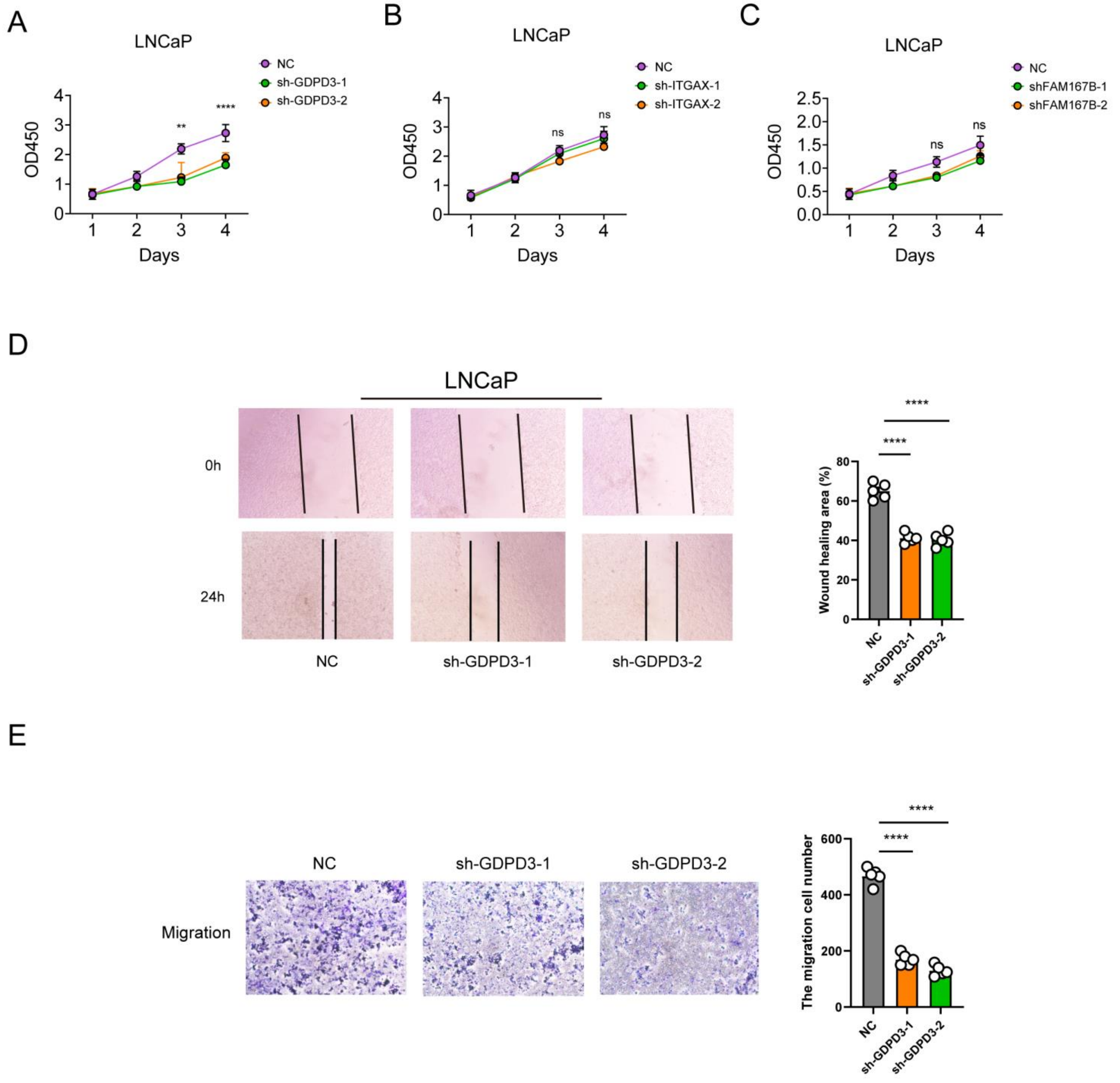

Figure. S9 Functional consequences of GDPD3, ITGAX, and FAM167B knockdown in LNCaP cells.

(A-E). Cell proliferation, migration, and invasion were assessed following knockdown of GDPD3, ITGAX, or FAM167B using shRNAs. Scale bar is 50 μm.

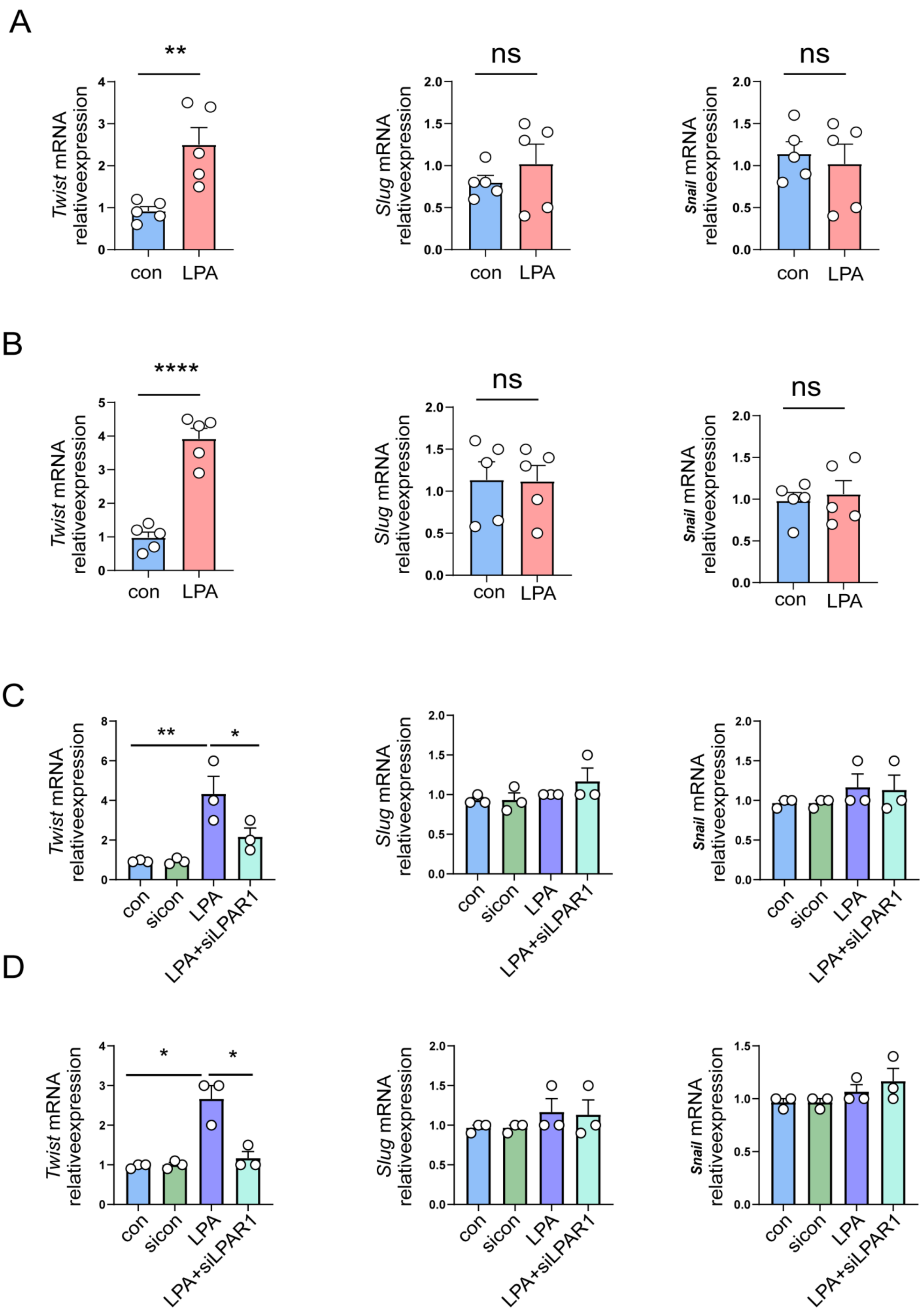
A
**
ns
ns
Twist mRNA relativeexpression
Slug mRNA relativeexpression
Snail mRNA relativeexpression
0
1
2
3
4
0.0
0.5
1.0
1.5
2.0
con
LPA
B
****
ns
ns
Twist mRNA relativeexpression
Slug mRNA relativeexpression
Snail mRNA relativeexpression
5
con
LPA
C
**
*
Twist mRNA relativeexpression
Slug mRNA relativeexpression
Snail mRNA relativeexpression
6
8
con
sicon
LPA
LPA+siLPAR1
D
*
*
Twist mRNA relativeexpression
Slug mRNA relativeexpression
Snail mRNA relativeexpression
con
sicon
LPA
LPA+siLPAR1

Figure. S10 The mRNA expression levels of the EMT regulators Twist, Slug, and Snail were assessed in DU145 and LNCaP cells.

(A, C) Relative mRNA expression of Twist, Slug, and Snail in DU145 cells measured by quantitative PCR. (B, D) Relative mRNA expression of Twist, Slug, and Snail in LNCaP cells measured by quantitative PCR. mRNA levels were normalized to GAPDH. Data represent mean ± s.d. from three independent experiments ($n = 3$). Statistical significance was determined using a two-tailed unpaired Student's *t*-test. Significance levels: $P < 0.05$ (*), $P < 0.01$ (**), and $P < 0.001$ (***).
