# Supplementary material for: Identify GDPD3 as a key regulator of epithelial–mesenchymal transition and prostate adenocarcinoma progression via the LPA/LPAR1/AKT axis: transcriptomic and experimental study
Source: Front Immunol. 2026 Jan 5;16:1637325. doi: 10.3389/fimmu.2025.1637325 (PMC12813044; doi:10.3389/fimmu.2025.1637325)
Supplement: Supplementary file 3 [file Table1.docx]

ITGAC

Forward Primer AGAGCTGTGATAAGCCAGTTCC

Reverse Primer AATTCCTCGAAAGTGAAGTGTGT

GDPD3

Forward Primer GCCAGTCGGGCCTAAACAG

Reverse Primer GTCCTCCAGACGAACCATGC

FAM167B

Forward Primer CTGCACCGACTGAAGATGGAC

Reverse Primer CTGATGTTCATGCGCGTGA

NDUFA4L2

Forward Primer ATGATCGGCTTAATCTGCCTG

Reverse Primer TCCGGGTTGTTCTTTCTGTCC

SDS

Forward Primer AGCGCCTCAAGAATGAAGGTG

Reverse Primer GGGGAATGTAGACCCAACCC 20

BRINP1

Forward Primer GACTGGCGTTGAGTACGTTGT

Reverse Primer TGATTTCGGCTGATCGACTGA

SMIS22

Forward Primer CACTCACCTTGTCATGGTATCAG

Reverse Primer CTGCTTCTTGGACTCAACTGC

PARM1

Forward Primer GTACAGAGTCTGCCTACATCAGC

Reverse Primer TGGAGAGCTAGTCCAGATGGT

GRIA4

Forward Primer TTCCGAGCAGCGTGCAAATA

Reverse Primer GCATTGGGGCTGGTGTTATGA

SCARF1

Forward Primer CCGATCAGACCTCAAGGACAG

Reverse Primer CCCAGGGTAGCTTGTGGGA

MT1A

Forward Primer AAATCATGGCACCTACTGTAGC

Reverse Primer CGGCGAATGAGAGAAGCCTC 20

CSF2

Forward Primer TCCTGAACCTGAGTAGAGACAC

Reverse Primer TGCTGCTTGTAGTGGCTGG

FAM107A

Forward Primer GCAGCGTGTCCTAGAGCAC

Reverse Primer CCGCAGGTTTTCCCTGACT

FREM2

Forward Primer CCTGCATGACCTGGTGTTG

Reverse Primer GCCAGTGCGTCGTTGTCTA

FERMTI

Forward Primer GCGTTGACCATCCCAATGAAG

Reverse Primer ACCAAAGAGCAAAGTCTGACC

KCTD16

Forward Primer ATGGCTCTGAGTGGAAACTGT

Reverse Primer TCAATGTGGAATGGCGAGTAAA

TACC1

Forward Primer AGGGGCAGTGATCTCCCAG

Reverse Primer TTTCTGACCACATGACGTGGA

SERPINB5

Forward Primer AATTCGGCTTTTGCCGTTGAT

Reverse Primer TGTCACCTTTAGCACCCACTT

SH3RF2

Forward Primer GGACGCCTGTGTTTTCCAAC

Reverse Primer TGAGCGCACTCCATCCAGA

PLEK2

Forward Primer GCGATGGTTCATCCTTCGG

Reverse Primer ATAGCCCCGGTGATCTCAAAG

PTACH

Forward Primer ACAAGACGGAACTGAGTGGTC

Reverse Primer GACACGTCCTTACATGGGTGA
